# Platinum(ii) complexes of aryl guanidine-like derivatives as potential anticancer agents: between coordination and cyclometallation†

**DOI:** 10.1039/d5ra00310e

**Published:** 2025-02-04

**Authors:** Patrick O'Sullivan, Viola Previtali, Brendan Twamley, Celine J. Marmion, Aidan R. McDonald, Isabel Rozas

**Affiliations:** a School of Chemistry, Trinity College Dublin 152-160 Pearse Street Dublin 2 Ireland rozasi@tcd.ie +353 1 896 3731; b Department of Chemistry, RCSI University of Medicine and Health Sciences 123 St. Stephen's Green Dublin 2 Ireland

## Abstract

The preparation of a wide variety of Pt(ii) complexes with aryl guanidines and their potential application as anticancer agents have been explored. A relatively facile synthesis of cyclometallated Pt(ii) complexes of arylguanidines, preparation of Pt(ii) guanidine coordination complexes and an *in situ* activation of platinum arylguanidine complexes with acetonitrile to create a bidentate aryl iminoguanidine Pt(ii) complex were achieved. Cyclometallation methodology was extended to create a water-stable conjugate incorporating two Pt(ii) ions and a diaryl bis-guanidine DNA minor groove binder. Several crystal structures were obtained confirming these complexation modes. The cyclometallated Pt(ii) complexes were particularly stable to aqueous environments and were tested for Reactive Oxygen Species generation and anticancer activity in a leukaemia cancer cell line.

## Abstract 2

The preparation of a wide variety of Pt(ii) complexes with aryl guanidines and their potential application as anticancer agents have been explored.

## Graphical abstract

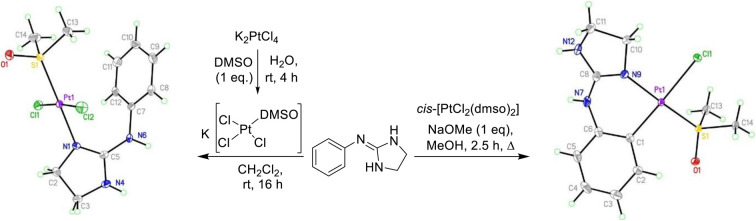

## Introduction

It is well known that platinum complexes like cis-platin and carboplatin play a crucial role in the treatment of several cancers. They exert their therapeutic activity by forming DNA adducts within cancer cells, thus inhibiting DNA replication and transcription and ultimately leading to cancer cell apoptosis or cell death.^1,2^ Platinum drugs are also widely employed in combination therapy regimens.^3,4^ Notwithstanding their success, platinum-based therapies also exhibit secondary adverse effects (myelosuppression, nephrotoxicity, ototoxicity, thrombocytopenia or extensive plasma protein binding) that limit their use and/or dose.^5^ The development of cancer drug resistance is also a major challenge. To overcome these unwanted shortcomings, new platinum-based derivatives are being continuously developed. These include but are not limited to non-classical Pt(ii) complexes, polynuclear Pt(ii) compounds, photoactivatable Pt(iv) complexes^6^ or multi-targeted Pt(ii) and Pt(iv) complexes, as summarised in recent comprehensive reviews.^7^ The multi-targeted approach has led to highly efficacious Pt(ii) complexes which not only target cancer cell DNA but also, amongst others, enzymes such as histone deacetylases, kinases, reductases, matrix metalloproteases and DNA topoisomerases, peptides and intracellular proteins such as STAT3 and tubulin. Examples also include Pt-DNA targeting agents that carry vectors such as sugars and hormone or integrin receptors to enable them selectively target cancer cells.

Among the non-classical Pt-based derivatives, Pt(ii) complexes of guanidine-like systems are particularly interesting. Guanidines have been shown to possess versatile biological properties with numerous reports of guanidine derivatives possessing anticancer, antibacterial, antifungal, antiprotozoal and antiviral activities and some guanidine-based drugs are already in clinical use, all of which are eloquently summarised by Roleira *et al.* in a 2023 comprehensive review.^8^ Guanidines are versatile ligands possessing a rich coordination chemistry. Different metal complexes with neutral guanidine, anionic guanidinate and dianionic guanidinate are known.^9,10^ For example, Jakupec and Keppler reported novel trans Pt complexes of guanidine synthesized by the nucleophilic addition of methylamine to di-alkyl cyanamide ligands of push–pull trans Pt nitrile complexes. Their *in vitro* assessment in different cancer cell lines indicated that the cytotoxicity of several *trans*-3,6 complexes was higher than that of *cis*-3,6 analogues. DNA interaction studies with some of these guanidine complexes confirmed that these compounds alter the DNA secondary structure, suggesting DNA as their possible target.^10^ Additionally, Carrillo-Hermosilla *et al.* published a very thorough review on the organometallic chemistry of guanidinate compounds, examining particular modes of coordination, reactivity and applications in catalysis or materials science.^11^ Thirupathi *et al.* reported in 2013 a series of interesting *cis*, *trans* and cycloplatinated guanidine Pt(ii) complexes. They achieved complexation of symmetrically tri-substituted aromatic guanidines using *cis*-[Cl_2_Pt(S(O)Me_2_)_2_] and NaOAc in refluxing methanol. Depending on the reflux time and nature of the substituents in the aryl systems, they achieved guanidine complexation or cycloplatination.^12^ Furthermore, Nieto *et al.* reported the reaction of a Pt(ii) reagent with ferrocene derivatives of substituted guanidines that produced the corresponding heterometallic complexes; further they proved that guanidine-based ferrocene–Pt complexes were active against different human cancer cells.^13^

Given the known propensity of the guanidium cation to form non-covalent interactions with the phosphate residues of DNA's minor groove helix and the fact that clinically used Pt(ii) drugs also target DNA albeit at a different site, namely by Pt binding to DNA nucleobases, we sought to explore the chemistry and biological activity of novel guanidine Pt(ii) complexes as a new class of anticancer agent. We had previously studied their suitability from a theoretical point of view,^14^ and found that the most favourable Pt(ii) complexes with guanidine derivatives were monodentate coordinated systems. Informed by these theoretical studies, our goal was to develop Pt(ii) complexes of aryl guanidine-like systems with the final aim to produce cytotoxic agents.

## Results and discussion

### Preparation of guanidine-like ligands

**Fig. 1:**
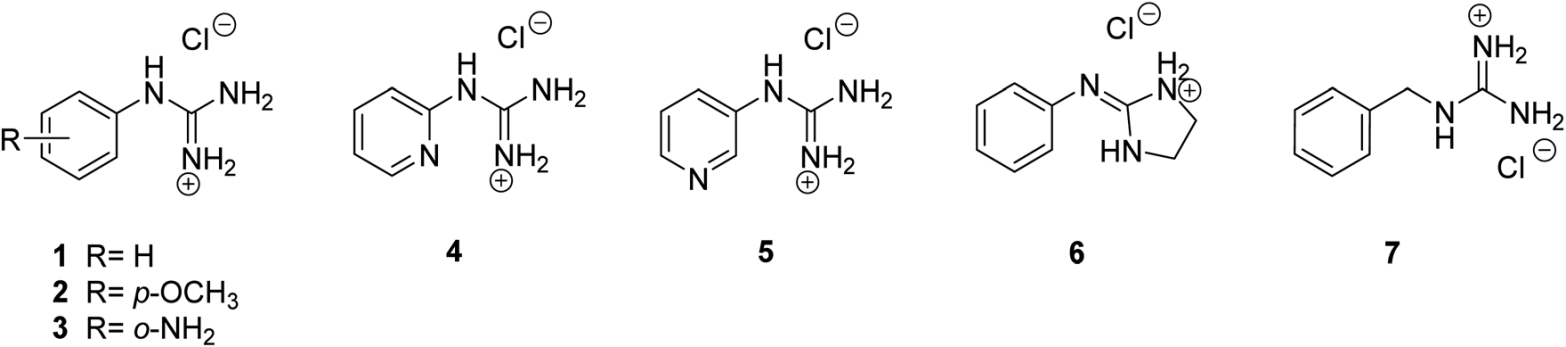
Structures of the guanidinium-like hydrochlorides 1–7, prepared as models to explore the conditions for the preparation of guanidine-Pt(ii) complexes.

To explore the best Pt complexation approach, mono-guanidine aryl systems (phenyl, pyridine, benzyl) with different substituents (*p*-OMe, *o*-NH_2_) were selected, not only to probe the electron density effect on the aryl ring, but also to obtain a diverse range of possible metal-binding domains, (*e.g.* pyridine N or *o*-NH_2_*vs.* guanidine/2-aminoimidazoline N atoms). Since complexation of guanidines with Pt may be carried out by direct reaction of the corresponding ligands with a Pt source, the corresponding aryl guanidine-based ligands were first synthesised (see details in ESI†). Thus, aryl guanidinium salts (1–5), 2-(iminophenyl)imidazolidinium (6) and benzylguanidinium (7) were prepared following standard methods (Fig. 1).

To prepare the corresponding Pt complexes, the conditions reported by Aitken *et al.*^15^ for the aminoguanidine complexation were followed by treating guanidinium salts 1 and 2 (5 eq.) with K_2_PtCl_4_ (1 eq.) in water in the absence of base. Precipitates, that were only soluble in DMSO, instantly formed corresponding to the tetrachloroplatinate salts of 1 and 2, clearly indicating that the use of the free base of these arylguanidinium salts was required in order to achieve Pt complexation. Accordingly, different bases (NH_4_OH, KO^*t*^Bu, KOH) in solutions of dichloromethane/water were explored to isolate the free base of 1 used as a model (Table 1). In all cases, the yields obtained were not ideal (0–66%, entries 1–3, Table 1) because the neutral guanidine was soluble in water. Considering that Yamada *et al.* had described the formation of a guanidine derivative in ethanol using sodium ethoxide as a base,^16^ and after adapting the procedure, the free base 8 was quantitatively obtained (entries 4–5, Table 1).

**Table 1:** Conditions screened for the generation of the free base of compound 1

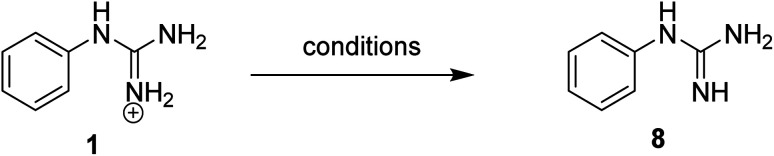			
Entry	Base	Solvent	Yield^a^
1	NH_4_OH (5 eq.)	CH_2_Cl_2_/H_2_O	0%
2	KO^*t*^Bu (5 eq.)	CH_2_Cl_2_/H_2_O	66%
3	KOH (pH 13)	CH_2_Cl_2_/H_2_O	41%
4	NaOEt (1.0 eq.)	EtOH	92%^b^
5	NaOEt (1.1 eq.)	EtOH	100%

a Isolated yields.

b Mixture of 8 and 1 (8%) as per ^1^H NMR.

**Scheme 1:**
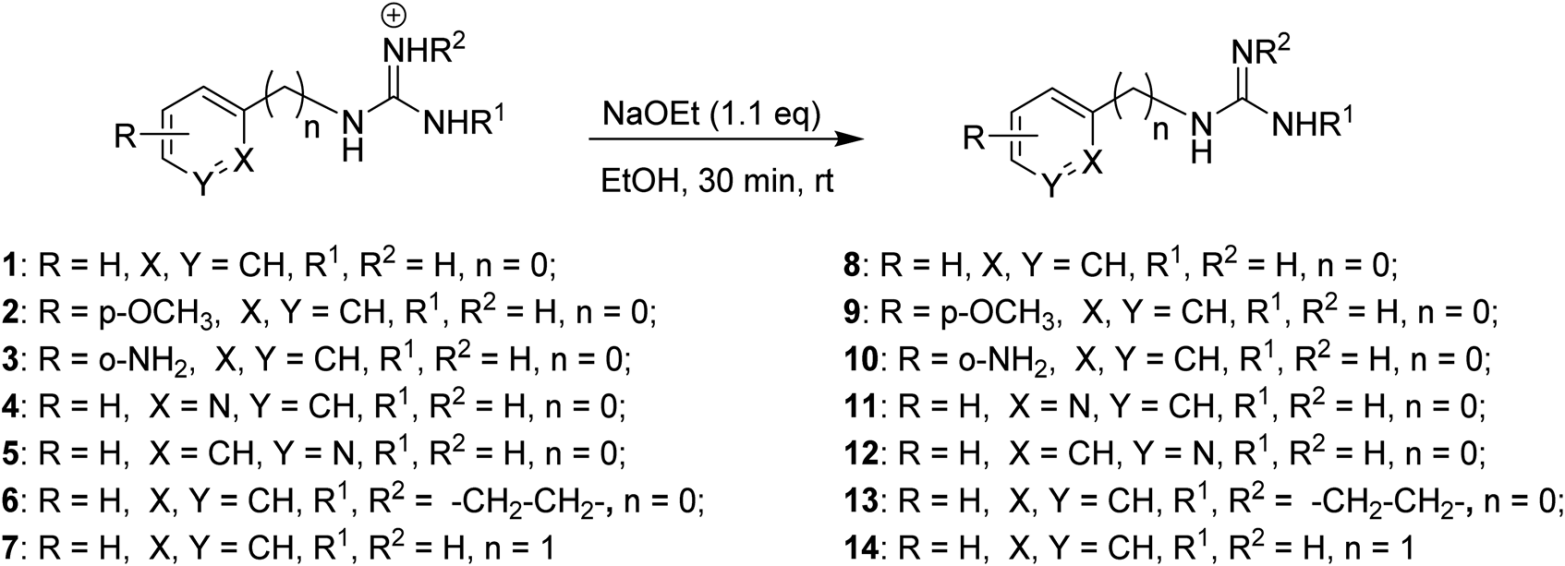
Aryl guanidinium/2-aminoimidazolinium derivatives converted to the corresponding aryl guanidine free base.

With these optimal conditions in hand (*i.e.*, 1.1 equiv. of sodium ethoxide in ethanol), conversion of the corresponding aryl guanidinium (1–5), 2-aminoimidazolinium (6) and benzyl guanidinium (7) salts to the corresponding free bases 8–14, was achieved with quantitative yields (Scheme 1).

Thus, seven guanidine-like ligands were prepared with the aim of forming the corresponding Pt(ii) complexes.

### Preparation of cyclometallated Pt(ii) guanidine complexes

Once these mono-guanidines were prepared, different approaches for their complexation with Pt(ii) were explored. First, commercial K_2_PtCl_4_ was reacted with phenylguanidine 8 in the presence of aqueous KOH at pH = 9 and pH = 13, at room temperature and at reflux, yielding in all cases a black precipitate of Pt(0) as the major product. Similarly, reaction of the free bases 8, 9 and 13 with K_2_PtCl_4_ in MeOH or H_2_O at room temperature in subdued lighting conditions led to decomposition to Pt(0). Next, considering that *cis*-[PtCl_2_(DMSO)_2_] had been reported to form Pt(ii) complexes with guanidine N atoms,^13,17,18^ this agent was tested to achieve complexation of the aryl guanidines prepared.

**Scheme 2:**
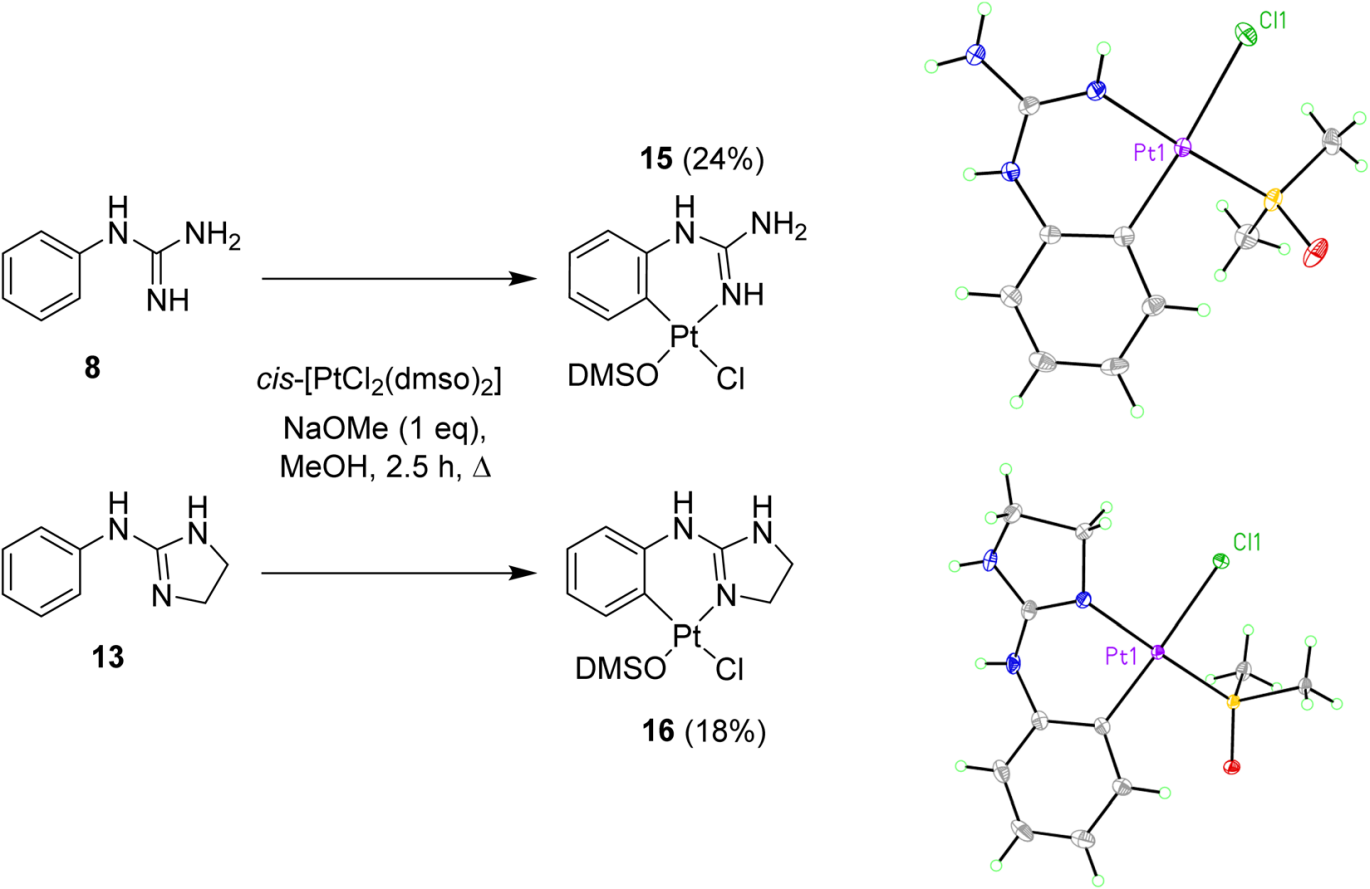
Preparation of cyclometallated Pt(ii) complexes 15 and 16 and crystal structures of the asymmetric unit in the corresponding complexes. Displacement ellipsoids are at 50% probability.

Complex *cis*-[PtCl_2_(DMSO)_2_] was prepared as previously described in the literature.^19^ Initial attempts of complexation using two equivalents of 8 and *cis*-[PtCl_2_(DMSO)_2_] in THF or chloroform at reflux resulted in the recovery of starting materials. When the reaction was carried out with one equivalent of 8 in methanol at reflux, two products were obtained, the cationic precursor of the starting material (*i.e.* compound 1) and a new derivative 15. After filtration and solvent removal *in vacuo*, the product was dissolved in the minimum amount of DMSO and precipitated out by addition of water. The solid obtained was characterised by NMR and IR spectroscopy. TOCSY and NOESY experiments, together with high temperature ^1^H NMR experiments of 15, showed Pt-complexation as indicated by satellite peaks at 6.3 ppm (NH) and 8.0 ppm (CH) (Fig. S1†). Additionally, the ^13^C NMR spectrum indicated coordination with DMSO and the IR spectrum showed an S–O stretch at 1091 cm^−1^. Furthermore, low resolution mass spectrometry revealed an [M + H]^+^ peak at 443.02, with a characteristic isotope splitting pattern for Pt and matching a calculated formula of [C_9_H_15_ClN_3_OPtS]^+^, thus suggesting that the fourth ligand must be Cl. Although there was no direct evidence of the arrangement of the Cl and DMSO ligands relative to the aryl guanidine, it was reasoned that DMSO would not bind *trans* due to the strong *trans*-effect of the cyclometallated group. Taking all this data into account a structure for the cyclometallated compound 15 was proposed (see Scheme 2). As mentioned, the reaction of one equivalent of free base 8 with excess of *cis*-[PtCl_2_(DMSO)_2_] produced cyclometallated product 15 and guanidinium salt 1. However, since a second equivalent of free 8 could act as a base and then generate a new molecule of free base 8 from the *in situ* formed salt 1, an equivalent of NaOMe was added to optimise the synthetic procedure, yielding exclusively the cyclometallated derivative 15 (Scheme 2). The reaction mixture was purified by repeated precipitation from a concentrated DMSO mixture with water and crystals of suitable quality for X-ray crystallography were grown in darkness at room temperature over a period of three months by slow evaporation of water into a concentrated DMSO solution of 15. Gratifyingly, the structure confirmed our solution phase assignment (see Scheme 2, Table S1 and details in ESI†).

Complex 15 crystallised in an orthorhombic unit cell (0.71 Å resolution); the relative configuration of the ligands was confirmed to be SP-4-4 (square planar 4-coordinate with the highest priority ligand, in this case Cl, *trans* to the fourth priority ligand, C). The strong *trans*-effect of C was demonstrated by the long Pt–Cl bond (2.4095(6) Å). The distances around the central guanidine C atom (C8) indicate that the double bond is localised between N10–C8 (1.306(3) Å) and that the other C–N bonds are single bonds in nature (*i.e.* N7–C8, 1.354(3) Å; and N9–C8, 1.350(3) Å). The N–Pt–C and Cl–Pt–S angles around Pt were 89.13(10)° and 92.57(2)° respectively, close to the ideal bond angle of 90° for a square planar complex. The crystal is stabilised by intermolecular hydrogen bonds (HBs) between the DMSO's O atom of one molecule and a H atom on the guanidine NH_2_ of another molecule.

A similar reaction using 2-aminoimidazolidine 13 produced the corresponding cyclometallated complex 16 (Scheme 2, Table S1 and details in ESI†) and, under identical conditions for crystallisation as those used with 15, suitable crystals for X-ray crystallography were formed and resolved (see Scheme 2). The crystal structure of 16 was very similar to that of 15; the Pt–Cl bond length was 2.4117(4) Å, and the N–Pt–C and Cl–Pt–S angles were 86.81(6)° and 87.785(15)°, and the unit cell was orthorhombic (0.71 Å resolution), in the space group *Pbca*. Like in complex 15, the distances around the central guanidine C atom (C8) indicate a localised double bond between N9–C8 (1.312(2) Å) while N7–C8 (1.345(2) Å), and N12–C8 (1.365(2) Å) seem to be single bonds. The main difference in the crystal packing was that 16 contained an intermolecular HB between the DMSO's O^1^ atom and the aniline-type N^7′^H of another molecule.

**Fig. 2:**
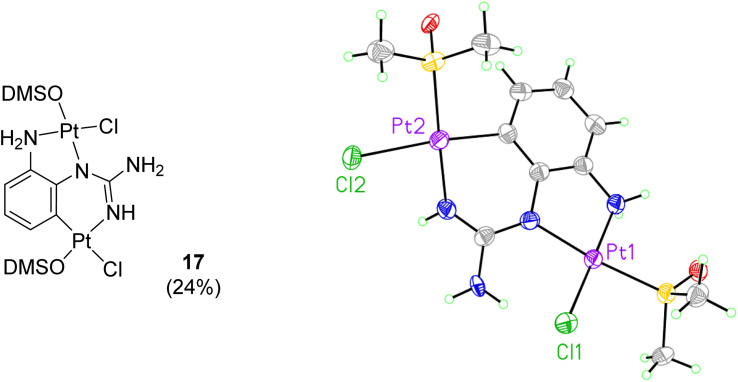
Crystal structure of bimetallic complex 17. Displacement ellipsoids are at 50% probability.

When reactions of *cis*-[PtCl_2_(DMSO)_2_] with *p*-methoxy phenylguanidine 9, pyridine guanidines 11 and 12, and benzylguanidine 14, were attempted in similar conditions, no products were obtained. However, the reaction with the *o*-amino phenylguanidine 10 yielded a bimetallic species (17, Fig. 2) In this complex, a combination of cyclometallated and non-cyclometallated interactions with each Pt(ii) centre is observed. Small purple crystals of suitable quality for X-ray diffraction were grown from a mixture of DMSO and H_2_O over a period of six weeks. Complex 17 crystallised in a monoclinic unit cell (*P*2_1_/*n* space group, 0.80 Å resolution, Fig. 2, Table S1 and details in ESI†). One of the Pt–Cl bonds is long, (2.421(3) Å) whereas the other is shorter (2.329(3) Å), again showing the strong *trans*-effect of C, with N–Pt–C and Cl–Pt–S angles of 85.4(4)° and 93.38(11)° respectively. Contrary to what was observed in complexes 15 and 16, distances around the central guanidine C atom (C8) point toward a delocalised double bond between N1–C7 (1.338(15) Å) and N3–C7 (1.313(14) Å), but this delocalization does not extend to N4–C7 (1.395(14) Å). When 17 was put in a solution of DMSO-*d*_6_ at room temperature, the compound degraded and was insoluble in any other solvent.

Once suitable conditions were found for the preparation of some phenyl guanidine-like Pt(ii) cyclometallated complexes we proceed to explore the requirements to prepare non-cyclometallated complexes.

### Preparation of coordinated Pt(ii) guanidine complexes

**Scheme 3:**
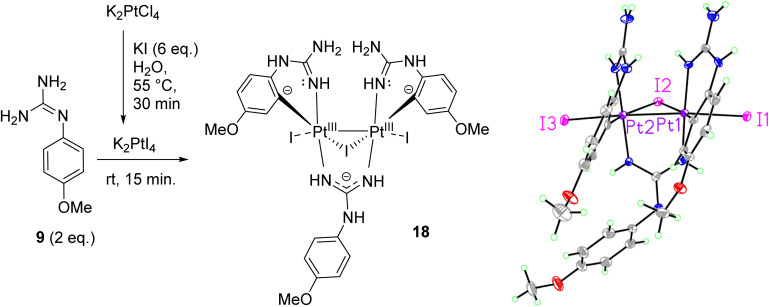
Preparation of dinuclear Pt(iii) complex 18 and crystal structure of the asymmetric unit. Displacement ellipsoids are at 50% probability.

Aiming to prepare non-cyclometallated Pt(ii) arylguanidine complexes, other Pt precursors were investigated. Thus, as in the synthesis of cis-platin, K_2_PtI_4_ was tested with the electron-rich *p*-methoxyphenyl guanidine 9. Accordingly, KI (6 eq.) was added to a solution of K_2_PtCl_4_ in H_2_O at 55 °C for 30 min to generate K_2_PtI_4_*in situ*.^20^ Then, free base 9 (2 eq.) was added at room temperature and the reaction stirred for 15 min. A brown precipitate was isolated by filtration and washed with water, dissolved subsequently in hot EtOAc, purified by slow diffusion of Et_2_O and then filtered. Crystals suitable for X-ray diffraction were grown from a standing solution of the filtrate to give 18 (Scheme 3, Table S1 and details in ESI†). The crystal structure of 18 (*P*2_1_/*c*, monoclinic, 0.78 Å resolution) was not the expected coordination complex and contained a mixture of cyclometallated and non-cyclometalated guanidine Pt interactions. This structure was notable for many features (see Scheme 3). First, the dinuclear structure [Pt_2_(*p*-OMe-PhGua)_3_I_3_] contained a Pt–Pt bond (2.6149(3) Å), with each Pt bound to one axial I atom (Pt–I = 2.7690(4) Å and 2.7412(4) Å) and one bridging I atom (Pt–I = 2.7967(4) Å and 2.8230(4) Å, Pt–I–Pt = 55.459(9)°). Moreover, each Pt atom was forming a cyclometallated complex with two different molecules of 9 (C–Pt–N = 91.97(19)° and 87.41(19)°), and there was also a bridging guanidinate group of a third 9 molecule (each Pt–N bond = 2.021(4), 2.024(4) Å).

There are two solvent pores in the crystal (Et_2_O and H_2_O). The three negatively charged guanidine and iodine ligands require a +6 overall charge; hence, considering the almost symmetrical nature of the complex, we can assume these two Pt centres to be Pt(iii). Most Pt(iii) complexes are bimetallic, stabilising the unpaired radicals in a Pt–Pt bond; besides, reported bimetallic Pt(iii) complexes have a distorted octahedral geometry, similar to that of 18.^21–23^ The ^1^H NMR in DMSO-*d*_6_ of 18 suggested that in solution the complex either decomposes or exists as a mixture of species.

**Scheme 4:**
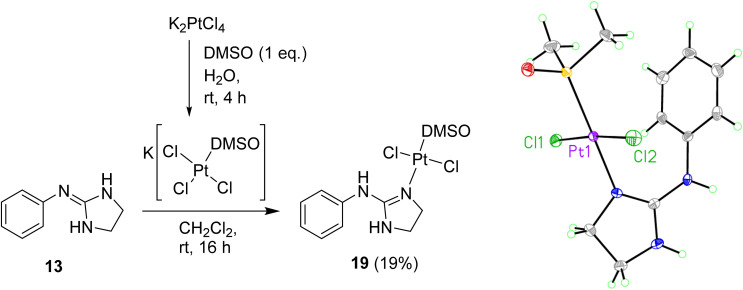
Preparation of Pt complex 19 and crystal structure of the asymmetric unit. Displacement ellipsoids are at 50% probability.

To prevent cyclometallation, milder conditions based on work by Kukushkin were attempted.^24,25^ Thus, K[PtCl_3_(DMSO)] was synthesised *in situ*, followed by addition of water-soluble ligand 13 and CH_2_Cl_2_ at room temperature to obtain *trans*-dichloro coordination/non-cyclometallated complex 19 after evaporation of the organic layer at room temperature and quick chromatography on silica (Scheme 4). Since pure 19 decomposed in DMSO, all ^1^H NMR experiments were carried out in DMF-*d*_7_ in which the complex is stable for at least seven days. The NOE experiments of compound 19 in DMF-*d*_7_ showed that no such effect existed between the DMSO protons and any other protons on the molecule, suggesting that DMSO is *trans* to the 2-aminoimidazoline ligand. High resolution mass spectrometry (ESI^−^) confirmed a molecular mass in agreement with the molecular formula C_11_H_17_Cl_2_N_3_OPtS. Crystals suitable for X-ray crystallography were grown over 12 h by slow diffusion of Et_2_O into a concentrated solution of 19 in CH_2_Cl_2_ (see Scheme 4, Table S1 and details in ESI†). The crystal structure of complex 19 again demonstrated that DMSO binds to Pt *trans* to the guanidine-like N. The compound crystallised in an orthorhombic unit cell (resolution = 0.71 Å) and *P*2_1_ 2_1_ 2_1_ space group. The crystal packing is stabilised by an intermolecular HB between the DMSO O^1^ of one molecule and the aniline-type N^6^H of another molecule. The Pt–Cl bond lengths are both 2.3101(10) and 2.3130(10) Å and the Cl–Pt–Cl angle is 174.65(4)° whereas the N–Pt–S angle is 179.19(10)°.

**Scheme 5:**
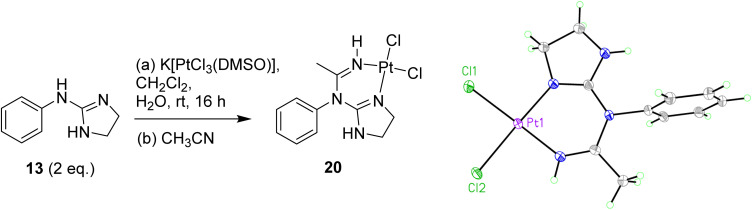
Preparation of Pt complex 20 with the insertion of an acetonitrile molecule and crystal structure of the asymmetric unit. Displacement ellipsoids are at 50% probability.

Exploring other conditions for the obtention of 19 in better yields, two equivalents of free base 13 were reacted with K[PtCl_3_(DMSO)] (Scheme 5), and when the crude reaction mixture from the organic layer was evaporated and re-dissolved in acetonitrile, crystals suitable for X-ray crystallography were isolated showing that, surprisingly, a reaction with a molecule of acetonitrile had taken place forming complex 20 (see Scheme 5, Table S1 and details in ESI†), which incorporates a methylimine into the structure.

The crystal structure of 20 is in a monoclinic unit cell (0.66 Å resolution), with space group *P*2_1_/*c*. The Pt–Cl and Pt–N bond lengths are 2.3104(5), 2.3105(5) Å and 1.9749(17), 1.9794(17) Å, and the N–Pt–N and Cl–Pt–Cl angles are 88.36(7)° and 88.959(18)°, respectively. The three C–N bond lengths around the guanidine C atom are 1.303(3) Å (N1–C5), 1.352(3) Å (N4–C5), 1.385(3) Å (C5–N6), and the C–N bond lengths around the amidine C atom are 1.385(3) Å (N6–C7) and 1.284(3) Å (C7–N8). Clearly, the C–N bonds coordinated to Pt are the shortest, implying that the guanidine/amidine double bonds are more localised on these atoms (the CN triple bond in the acetonitrile solvate is much shorter, 1.13 Å). The other three C–N bonds to the guanidine or amidine C atom are intermediate in length between single and double bonds, demonstrating some degree of resonance. The C–N bonds to the imidazole CH_2_ groups (1.453(3) Å and 1.476(3) Å) and to the aryl ring (1.452(2) Å) are clearly single bonds.

Regarding the possible mechanisms for the acetonitrile insertion, it is not clear whether the acetonitrile first coordinates to K[PtCl_3_(DMSO)] and then undergoes nucleophilic addition to coordinated 2-aminoimidazoline, if the coordinated acetonitrile is attacked by free 2-aminoimidazoline or if coordination of both ligands first takes place followed by C–N bond formation. Attack of coordinated 2-aminoimidazoline on uncoordinated acetonitrile was ruled out for steric reasons and the lack of precedence in the literature for nucleophilic attack on inactivated nitriles. In contrast, the strongly electrophilic properties of metal-acetonitrile complexes have been comprehensively reviewed.^26^

Further attempts to prepare more non-cyclometallated complexes like 19 with the rest of the free bases were unsuccessful as these compounds did not dissolve in the biphasic H_2_O/CH_2_Cl_2_ solvent system at room temperature as used in Scheme 4. Thus, a single solvent system was attempted using either MeOH or THF with isolated K[PtCl_3_(DMSO)]; however, these reactions gave a mixture of many compounds as adjudged by ^1^H NMR.

Considering the results obtained so far, the cyclometallation of in-house diaryl bis-guanidine systems known to be good DNA minor groove binders was next attempted.

### Preparation of cyclometallated complexes of bis-guanidine diaryl systems

**Scheme 6:**
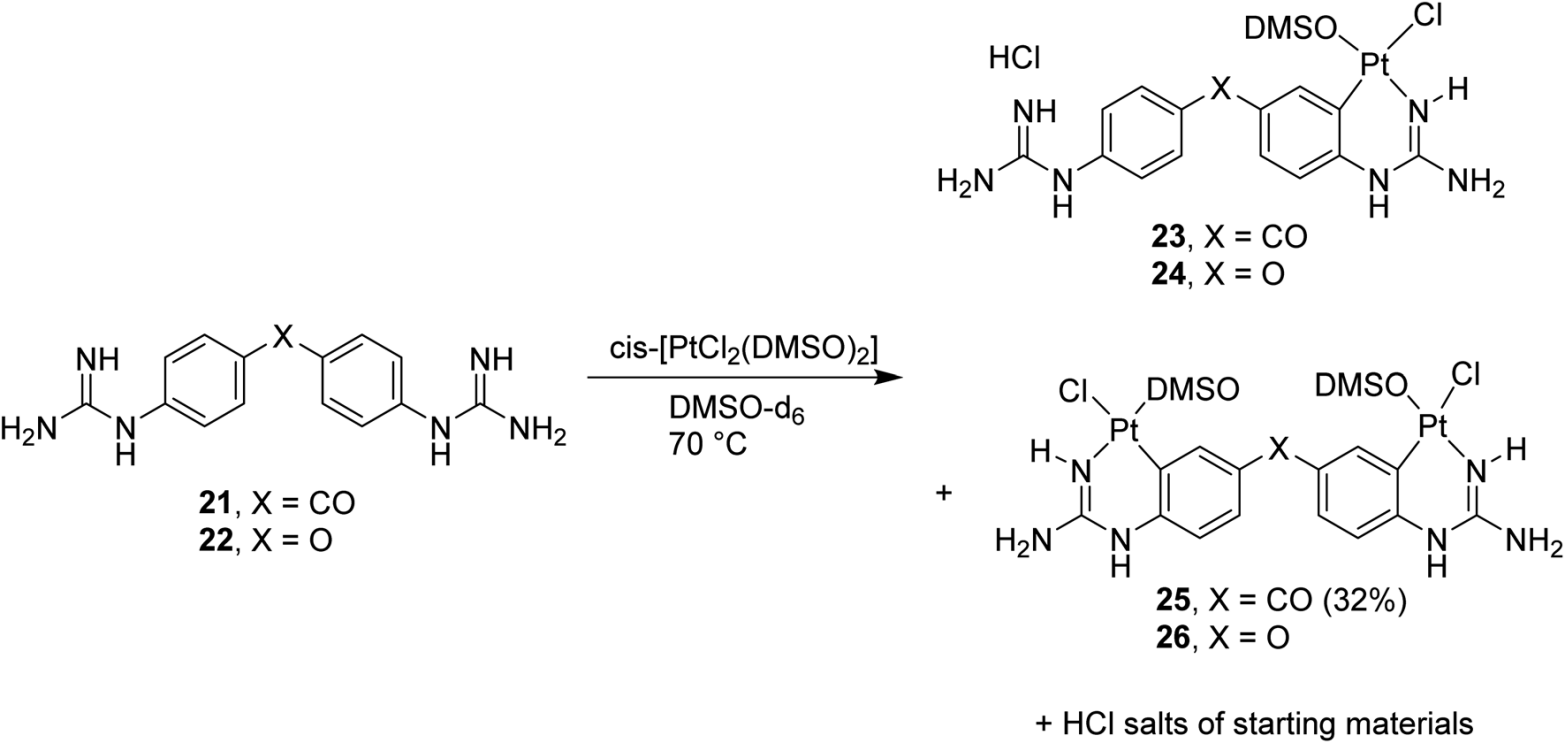
Cyclometallation reactions with bis-guanidine diaryl systems 21 (X = CO) and 22 (X = O) followed by ^1^H NMR.

Once the conditions for Pt cyclometallation of mono-guanidine-like systems 8 and 13 were established, the corresponding complexation of two bis-guanidinium systems, previously prepared in our lab showing good binding to the minor groove of DNA, was attempted. Thus, the free bases 21 and 22 were prepared and exposed to the corresponding cyclometallation conditions (*i.e. cis*-[PtCl_2_(DMSO)_2_], NaOCH_3_, CH_3_OH, 70 °C, 2 h; Scheme 6); however, only an insoluble yellow precipitate was formed in both cases instead of cyclometallation. Free bases 21 and 22 have two N atoms capable of forming bis-Pt complexes by coordinating with one N atom of another ligand and, hence, these potential oligomers could keep polymerising yielding the precipitate observed. To stop this hypothesised polymerisation and to identify the monomer complex, NaOCH_3_ was removed, the solvent changed to DMSO-*d*_6_, and the reaction with 21 was followed by ^1^H NMR as indicated in the spectra shown in Fig. S2† confirming the formation of a mixture of the hydrochloride salt of the starting material, mono- and di-cyclometallation product.

Thus, in the absence of MeOH or NaOCH_3_ both reactions yielded the mentioned mixture as detected by NMR; however, isolation of the complexes proved difficult since all three species for each reaction were water soluble except for compound 25 (Scheme 6). Repeated dissolution of the crude mixture in DMSO, followed by precipitation with H_2_O and filtration gave a filtrate from which carbonyl-linked 25 crystallised. Despite repeated attempts, purification of 23, 24 or 26 did not yield adequate material for characterisation.

**Fig. 3:**
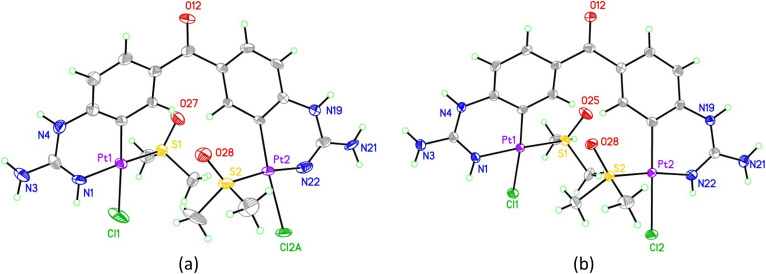
(a) Asymmetric unit of the first crystal structure obtained of 25 showing the majority occupied disordered moiety (Pt2, Cl2A). (b) asymmetric unit of the second crystal structure obtained of 25. In all structures displacement ellipsoids are at 50% probability. See ESI† for structures including solvents/disorder.

The X-ray structure of 25 (Fig. 3a, Table S1 and details in ESI†) was in agreement with the solution structure. The binuclear complex crystallised in an orthorhombic unit cell (0.80 Å resolution, *Pna*2_1_ space group). Restraints and constraints were used to model the disorder in one of the Pt–Cl positions (72%/28% occupancy). There are three and a half water molecules per asymmetric unit.

Aiming to improve the preparation of cyclometallated Pt complexes of bis-guanidines 21 and 22, many variations of the biphasic reaction from Scheme 5 were attempted, but no product could be isolated. Switching to a mono-phasic system using MeOH with K[PtCl_3_(DMSO)] and free base 21, yielded again cyclometallated 25 after crystallisation from aqueous solution and a new sample suitable for X-ray diffraction was solved (Fig. 3b, Table S1 and details in ESI†). In this case, 25 crystallised in a triclinic unit cell (*P*1̄ space group, 0.78 Å resolution) with two water molecules per complex. The Pt–N bonds were 1.995(4) and 2.008(4) Å. The Pt atoms were both square planar. Within the platinacycles, the C–Pt–N angles were both 89° (89.12(18) and 88.79(17)°). However, the C–Pt–S angle was 98° (98.44(14) and 97.91(13)°) in both cases. The overall molecule showed a helical twist. The Pt–Pt distance was *ca.* 6.09 Å and the angle between the planes of the square planar Pt geometries was 37.3(1)°. The dihedral angle between the planes of each benzene ring was 49.3(2)°.

In summary, bis-guanidine diaryl systems are likely to cyclometallate, even at room temperature and, therefore, non-cyclometallated Pt-complexes bound *via* a guanidine, even if isolated from a reaction mixture, would likely decompose under biological conditions.

### Assessment of cytotoxic potential of the cyclometallated complexes

#### Reactive oxygen species generation

**Fig. 4:**
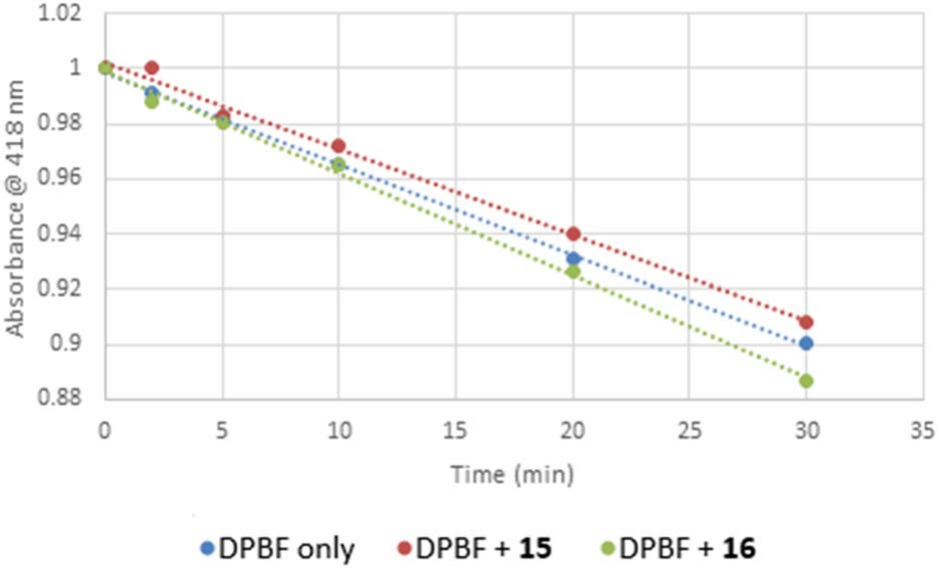
Graph of change in DPBF absorbance *vs.* time in the presence of DMSO (blue dots, slope = −0.0033 min^−1^), with 15 (red dots, slope = −0.0031 min^−1^) and with 16 (green dots, slope = −0.0037 min^−1^). All linear fits (*R*^2^ = 0.999). Each data point is an average of two runs.

Considering that Pt-cyclometallated complexes have been shown to generate singlet oxygen (^1^O_2_),^27^ the potential Reactive Oxygen Species (ROS) generation for complexes 15 and 16 was investigated as a potential cytotoxic effect. The strongly absorbing compound 1,3-diphenyl-2-benzofuran (DPBF, *λ*_max_ = 418 nm in DMSO) is known to react with ^1^O_2_ destroying the chromophore and, therefore, the decrease in absorbance at 418 nm can be used as a measure of how much ^1^O_2_ is produced.^28^ To avoid the background reaction of light with triplet oxygen (^3^O_2_) producing ^1^O_2_ in an open air atmosphere,^29^ white light was used at an intensity that decreased the intensity of DPBF (100 mM) absorption at 418 nm by 10% after 30 min (Fig. S3a†). This ensured that enough light was present to generate ^1^O_2_, but not too much that it would mask ^1^O_2_ generation from the complexes under investigation. The experiment was run in DMSO to ensure that 15 and 16 (both 150 μM) were fully solubilised. Fig. S3a–c† show that the decrease in absorbance at 418 nm was very similar in either the presence (Fig. S3b and c†) or absence (Fig. S3a†) of the Pt complexes, and the combined normalised results are shown in Fig. 4. Each compound shows a linear degradation of DPBF over time. All three systems and conditions (DPBF alone, DPBF with 15 and DPBF with 16) gave very similar results. The slope of DPBF alone (−0.0033 min^−1^) and with 15 (−0.0031 min^−1^) are almost identical. The slope of DPBF with 16 (−0.0037 min^−1^) gives a measurably, albeit very slight improvement in ^1^O_2_ generation. From this data, we can conclude that for therapeutic purposes both 15 and 16 should be considered inactive as ROS generators.

#### Cancer cell viability studies

Finally, to measure the effect of Pt-based systems 15, 16, 17 and 35 in the cell viability of cancer cells, preliminary investigations utilising the AlamarBlue® assay were explored using the HL-60 leukaemia cell line (see details in ESI†). These compounds were dissolved in DMSO in a stock solution and tested in cells at a final concentration of 0.1% DMSO in buffer. Since these compounds crystallised out of DMSO/H_2_O solutions over a period of months, were stable for at least one year in DMSO-*d*_6_ at room temperature (as adjudged by ^1^H NMR) and could be heated to 80 °C in DMSO-*d*_6_ and cooled to room temperature without a noticeable change in the ^1^H NMR spectrum, we were confident that DMSO would not interfere with the cell viability experiments over three days at 37 °C. The calculated IC_50_ values using 10 000 and 40 000 cells per well are shown in Table 2. Binuclear complex 25 had weak but still measurable cytotoxicity with 10 000 cells per well assays (IC_50_ = 101 μM); however, this is lost when running the experiments with 40 000 cells per well (IC_50_ > 100 μM). The cycloplatinated 2-aminoimidazoline complex 16 was the most active (IC_50_ = 55 μM in 10 000 cells per well and IC_50_ = 65 μM in 40 000 cells per well) indicating that the five-membered 2-aminoimidazoline ring may be important for activity. Furthermore, the cytotoxicity of Pt complex 16 compares well to the bioactive mono- and bis-isouronium and bis-hydroxyguanidinium families previously tested in our group.^30^

**Table 2:** IC_50_ values calculated for Pt complexes in HL-60 cells using the AlamarBlue® assay

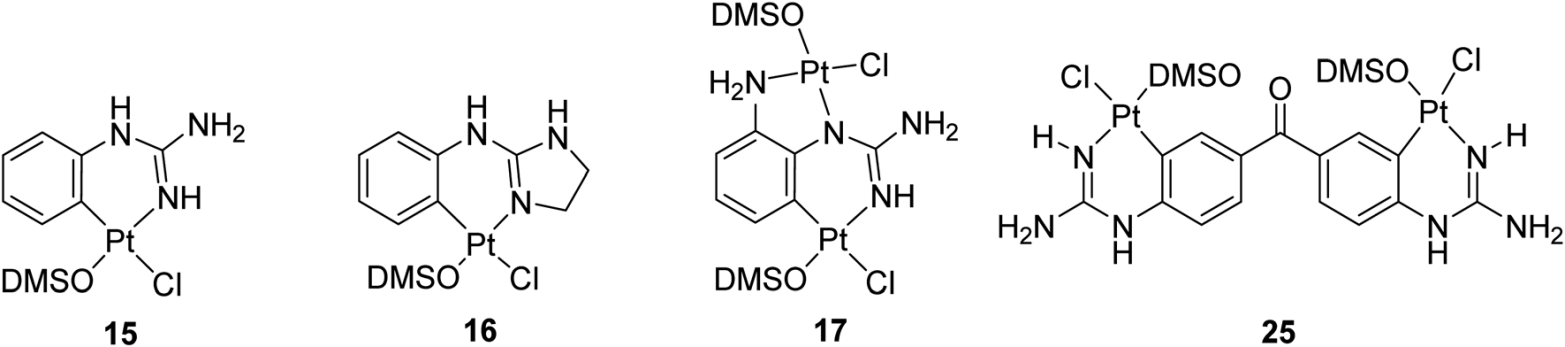		
Compd	IC_50_ ± SEM^a^ (μM) 10 000 cells per well	IC_50_ ± SEM^b^ (μM) 40 000 cells per well
15	>100	>100
16	55.29 ± 3.0	66.82 ± 5.9
17	100.81 ± 3.4	>100
25	>100	>100
Carboplatin	12.62 ± 1.7	N/A

a Cells were seeded at a density of 5 × 10^4^ cells per mL in a 96-well plate and treated with the compounds dissolved in 0.1% DMSO in ddH_2_O at 10 μM, 25 μM, 50 μM, 75 μM and 100 μM. Carboplatin (dissolved in ddH_2_O) was used as a reference and tested in the same manner. Once treated, cells were incubated for 72 h at 37 °C after which they were treated with AlamarBlue® and left in darkness in an incubator for 5 h. The resulting fluorescence was read using a plate reader from which percentage viability was calculated. IC_50_ values were calculated using Prism GraphPad 5 software from at least three independent experiments performed in triplicate.

b As note (a) but cells were seeded at a density of 2 × 10^5^ cells per mL.

It is interesting to note that the free ligand in complex 25 (*i.e.* compound 21) had previously been shown to have no activity in HL-60 cells;^31^ however, complexation with Pt resulted in a weak though measurable effect on cell viability.

## Experimental

### Synthesis

#### Preparation of cycloplatinated arylguanidine and 2-arylaminoimidazoline complexes

A suspension of *cis*-[PtCl_2_(DMSO)_2_] (1.0 eq., 0.888 mmol), the appropriate free base (1.0 eq., 0.888 mmol) in DMSO (1 mL) or in a fresh NaOMe solution (1.1 eq., 1.00 mmol in MeOH, 24 mL) was heated to 80 °C for 2–48 h. MeOH was removed *in vacuo* and the solid was dissolved in a minimum of DMSO (*ca.* 1 mL), filtered through cotton wool and precipitated using H_2_O (5–30 mL). The fine solid was collected by centrifugation (8000 rpm), re-dissolved in DMSO (0.2 mL) and precipitated with H_2_O if necessary. The powder was dried at rt *in vacuo* overnight at *ca.* 10 mbar.

#### *a*-Chlorido-*b*-(DMSO-*S*)-*cd*-(2-phenylyl-*κC*^*2*^-guanidine-*κN*)platinum(ii) (15)

Free base 8 (119 mg, 0.888 mmol) and *cis*-[PtCl_2_(DMSO)_2_] (360 mg, 0.888 mmol) were added to a freshly prepared NaOMe solution produced from Na metal (24 mg, 1.00 mmol) and MeOH (24 mL), affording after precipitation the title compound as a yellow solid (94 mg, 24%). Crystals suitable for XRD were grown over three months by slow evaporation of H_2_O into a concentrated solution of the title compound in DMSO. *δ*_H_ (400 MHz, DMSO-*d*_6_): 3.35 (s, 6H, CH_3_), 6.19 (br s, 1H, PtNH), 6.31 (br s, 2H, NH_2_), 6.59–6.72 (m, 2H, Ar-3 & Ar-4), 6.96 (t, 1H, J 7.4, Ar-5), 7.97 (d + dd, 1H, J 7.7 + J 62.6, Ar-6), 9.13 (br s, 1H, ArNH). *δ*_C_ (100 MHz, DMSO-*d*_6_): 46.2 (CH_3_), 113.1 (q Ar-1), 115.0 (CH Ar-3), 121.2 (CH Ar-4), 124.1 (CH Ar-5), 137.0 (q Ar-2), 138.3 (CH Ar-6), 150.4 (q C=N). *δ*_Pt_ (86 MHz, DMSO-*d*_6_): −3582. *ν*_max_ (ATR)/cm^−1^: 3407 (NH), 3310 (N), 3193 (NH), 2352, 1645 (C=N), 1602, 1551, 1479, 1399, 1300, 1091 (S–O), 1022, 758, 721. % Calculated for C_9_H_14_N_3_OPtClS·0.5NaCl·1.5H_2_O·0.5DMSO: C 22.32, H 3.28, N 7.81. % Found: C 22.12, H 3.01, N 8.01. LRMS (*m*/*z* ESI^+^) found: 444.2 ([M + H]^+^. C_9_H_15_N_3_OSCl_2_Pt requires 444.03).

#### *a*-Chlorido-*b*-(DMSO-*S*)-*cd*-([2-aminophenylyl]-*κC*^*2*^-imidazoline-*κN*)platinum(ii) (16)

Free base 13 (200 mg, 1.15 mmol) and *cis*-[PtCl_2_(DMSO)_2_] (462 mg, 1.15 mmol) were added to a freshly prepared NaOMe solution produced from Na metal (29 mg, 1.18 mmol) and MeOH (25 mL), affording after precipitation the title compound as a dark blue solid (90 mg, 18%). M.P. 223–224 °C. Crystals suitable for XRD were grown over three months by slow evaporation of H_2_O into a concentrated solution of the title compound in DMSO. *δ*_H_ (400 MHz, DMSO-*d*_6_): 3.14–3.57 (m, 8H, 2 × CH_3_ & CH_2_), 3.99 (br s, 2H, CH_2_), 6.37–6.77 (m, 2H, Ar-3 & Ar-4), 6.77–7.11 (m, 1H, Ar-5), 7.11–7.57 (m, 1H, NH), 7.57–8.01 (m, 1H, Ar-6), 9.53 (br s, 1H, NH). *δ*_C_ (100 MHz, DMSO-*d*_6_): 44.0 (CH_2_), 46.1 (CH_3_), 51.5 (CH_2_), 114.9 (q Ar-1), 115.0 (CH Ar-4), 121.8 (CH Ar-3), 124.6 (CH Ar-5), 137.8 (q Ar-2), 139.6 (CH Ar-6), 156.0 (q C=N). *δ*_Pt_ (86 MHz, DMSO-*d*_6_) −3633. *ν*_max_ (ATR)/cm^−1^: 3339, 3282 (NH), 3185 (NH), 2994 (CH), 1606, 1578, 1465, 1413, 1288, 1094, 1017, 743. % Calculated for C_11_H_17_N_3_OPtClS·H_2_O·0.5DMSO: C 27.35, H 3.73, N 7.97. % Found: C 27.16, H 3.45, N 8.16.

#### Bis–Pt complex of 2-aminophenylguanidine (17)

Free base 10 (15 mg, 0.1 mmol) and *cis*-[PtCl_2_(DMSO)_2_] (20 mg, 0.05 mmol) were dissolved in DMSO-*d*_6_ (1 mL), affording after precipitation the title compound as a dark purple solid (6 mg, 24%). Crystals suitable for XRD were grown over three months by slow evaporation of H_2_O into a concentrated solution of the title compound in DMSO.

#### Triiodo-tris(4-methoxyguanidine)bis-platinum(iii) complex (18)

To K_2_PtCl_4_ (41 mg, 0.1 mmol) in H_2_O (1 mL) at 60 °C, was added KI (100 mg, 0.6 mmol). The mixture was stirred in the dark for 20 min, after which free base 9 (37 mg, 0.2 mmol) was added. The yellow mixture was stirred at r.t. for 15 min and the brown precipitate was filtered and washed with H_2_O. The compound was dissolved in EtOAc and crystallised out by slow evaporation of hexane to give crystals suitable for XRD. No further characterisation data is available for this compound.

#### *trans*-[Dichloro(DMSO)(2-[phenylamino]imidazoline-*κN*)platinum] (19)

To a solution of K_2_PtCl_4_ (139 mg, 0.335 mmol) in H_2_O (0.5 mL) was added dropwise a solution of DMSO (24 μL, 0.335 mmol) in H_2_O (0.5 mL) and the reaction was stirred at r.t. for 4 h until the colour changed from red to yellow. The solid free base 13 (54 mg, 0.335 mmol) and CH_2_Cl_2_ (0.5 mL) were then added, and the reaction was stirred at r.t. for 16 h. The organic layer was separated and evaporated at r.t. by blowing with Ar to give a crude oil which was purified on silica, eluting in 0.5% acetone in CH_2_Cl_2_ to give the title compound as a yellow solid (33 mg, 19%). Crystals suitable for XRD were grown over 4 h by slow evaporation of Et_2_O into a concentrated solution of the title compound in CH_2_Cl_2_. *δ*_H_ (400 MHz, DMF-*d*_7_): 3.37 (s, 6H, CH_3_), 3.59 (t, 2H, J 9.1, CH_2_), 3.85 (t, 2H, J 9.1, CH_2_), 7.15 (br s, 1H, NH), 7.20 (t, 1H, J 7.4, *p*-Ar), 7.30 (d, 2H, J 7.5, *o*-Ar), 7.38–7.43 (m, 2H, *m*-Ar), 8.88 (br s, 1H, NH). *δ*_C_ (100 MHz, DMF-*d*_7_): 42.5 (CH_3_), 43.5 (CH_2_), 51.4 (CH_2_), 123.1 (CH *o*-Ar), 124.8 (CH *p*-Ar), 129.4 (CH *m*-Ar), 138.6 (q Ar), 160.7 (q C=N). *δ*_Pt_ (86 MHz, DMF-*d*_7_): −3033. *ν*_max_ (ATR)/cm^−1^: 3289 (NH), 3090 (NH), 1617 (C=N), 1093 (S–O), 3018, 1588, 1571, 1478, 1432, 1267, 1022, 730. HRMS (*m*/*z* ESI^−^) Found: 503.0063 ([M − H]^−^. C_11_H_16_N_3_OSCl_2_Pt requires 503.0039).

#### *cis*-[Dichloro(phenyliminoguanidine (*N*,*N*′))platinum] (20)

To a crude mixture of 19 prior to column chromatography was added CH_3_CN (1 mL). Crystals suitable for XRD were grown over a period of months from this standing solution. *δ*_H_ (400 MHz, DMF-*d*_7_): 3.52–3.58 (m, 2H, CH_2_), 4.25–4.33 (m, 2H, CH_2_), 6.55 (br s 1H, NH), 7.75–7.81 (m, 3H, Ar), 7.86–7.90 (m, 2H, Ar), 10.04 (br s, 1H, NH). NOTE: CH_3_ obscured by H_2_O peak. *δ*_C_ (100 MHz, DMF-*d*_7_): decomposed over time of experiment (v dilute). *ν*_max_ (ATR)/cm^−1^: 3390, 3254 (NH), 2350, 2164 (CN), 1622(C=N), 1576, 1436, 1271, 1098, 1030, 730.

#### 4,4′-Bis-[*a*-chlorido-*b*-(DMSO-*S*)-*cd*-(2-phenylyl-*κC*^2^-guanidine-*κN*)platinum(ii)]ketone (25)

Free base 21 (15 mg, 0.05 mmol) and *cis*-[PtCl_2_(DMSO)_2_] (40.5 mg, 0.1 mmol) dissolved in DMSO-*d*_6_ (1 mL) at 80 °C for 48 h, afforded after precipitation the title compound as a yellow solid (16 mg, 32%). M.p. 250 °C. Crystals suitable for XRD were grown over three months by slow evaporation of H_2_O into a concentrated solution of the title compound in DMSO. *δ*_H_ (400 MHz, DMSO-*d*_6_): 3.36 (s, 12H, CH_3_), 6.40 (d, 2H, J 2.5, PtNH), 6.43 (s, 4H, NH_2_), 6.75 (d, 2H, J 8.2, Ar-5), 7.38 (dd, 2H, J 1.9, J 8.2, Ar-6), 8.48 (d, 2H, J 1.9, Ar-2), 9.48 (d, 2H, J 2.5, NH). *δ*_C_ (150 MHz, DMSO-*d*_6_): 46.0 (CH_3_), 112.2 (q Ar C-3), 114.4 (Ar C-5), 126.4 (Ar C-6), 131.0 (Ar C-1), 140.3 (q Ar C-4), 140.8 (Ar C-2), 150.1 (q C=N), 194.4 (q C=O). *δ*_Pt_ (86 MHz, DMSO-*d*_6_): −3556, −3559. *ν*_max_ (ATR)/cm^−1^: 3356 (NH), 3206 (NH), 3000, 2917 (CH), 1649, 1619, 1535, 1477, 1304, 1288, 1249, 1093, 1017, 1002, 947, 823, 748, 677. % Calculated for C_19_H_26_N_6_O_3_Pt_2_Cl_2_S_2_·6NaCl·6H_2_O·4DMSO: C 19.27, H 2.55, N 4.99. % Found: C 19.32, H 2.90, N 5.21.

### ROS generation experimental details

For the evaluation of the singlet oxygen production, solutions the ^1^O_2_ trap 1,3-diphenylisobenzofuran (DPBF) in DMSO were employed (20 mg in 50 mL). Calibration was achieved using 50 : 50 of a 112 μM DPBF solution to get absorption 1 at *λ*_max_ = 418 nm (in DMSO). Next, a fresh solution of each Pt complex (15, or 16) in DMSO was prepared at high concentration (150 μM), added to the cuvette and its absorbance was adjusted to around 0.01 at wavelength of irradiation. Potential singlet oxygen production was assessed looking at the decay absorption spectra of DPBF at time points 0, 2, 5, 10, 20, and 30 min. Since no clear effect was observed in the presence of the DMSO Pt(ii) complexes solutions, no further measurements at a lower concentrations were carried out. The slope of plots of absorbance of DPBF at 418 nm *vs.* irradiation time for each photosensitizer was calculated.

### Cell viability experimental details

The HL-60 (human caucasian promyelocytic leukemia) cell line was maintained between 200 000–2 000 000 cells per mL in Roswell Park Memorial Institute (RPMI) 1640 medium with stable glutamate (GlutaMax I) supplemented with 10% (v/v) foetal bovine serum (FBS) and 50 μg per mL penicillin/streptomycin (pen-strep). The growth medium was stored in the fridge at 4 °C and heated to 37 °C prior to culture work. Cells were grown at 37 °C in a humidified environment maintained at 95% O_2_ and 5% CO_2_ and passaged at least three times weekly depending on their levels of confluency. When required for sub-culturing, cells were transferred to a sterile tube and centrifuged at 1296 rpm for 5 min. The supernatant was discarded, and the cell pellet was resuspended in fresh medium. Cells were then counted using a haemocytometer slide and seeded at the required density.

HL-60 cells in the log phase of growth were seeded in 96-well plates at a density of 50 000 cells per mL (200 μL per well or 10 000 cells per well) in complete RPMI medium the same day of the experiment. Compounds 13, 16, 17 and 25 were dissolved in DMSO to obtain a starting 100 mM stock solution. The cells were then treated with either 2 μL of a 1 : 100 dilution of stock concentrations of drugs or ddH_2_O as vehicle control, or 0.2 μL of a 1 : 1000 dilution of stock concentrations of drugs or DMSO as vehicle control. All experiment were repeated in triplicate for at least three times. Three wells containing 200 μL RPMI with no cells were also set up as blanks.

After 72 h incubation, 20 μL AlamarBlue® was added to each well. The plates were incubated in darkness at 37 °C for 5 hours. Using a Molecular Devices microplate reader, the fluorescence (*F*) was then read at an excitation wavelength of 544 nm and an emission wavelength of 590 nm. Cell viability was then determined by subtracting the mean blank fluorescence (*F*_b_) from the treated sample fluorescence (*F*_s_) and expressing this as a percentage of the fluorescence of the blanked vehicle control (*F*_c_). This is demonstrated in the equation below. The results were then plotted as nonlinear regression, sigmoidal dose–response curves on Prism GraphPad 5 software, from which the IC_50_ value for each drug was determined.
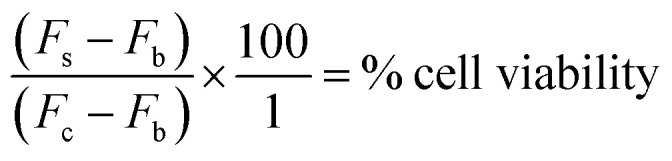


## Conclusions

The preparation of Pt(ii) complexes of aryl guanidines has been explored. Suitable conditions have been found for the preparation of unsubstituted phenyl guanidine-like Pt(ii) cyclometallated complexes. However, replacement of the phenyl system by a pyridine, separation of the guanidine and phenyl moieties by a methylene group or *p*-substitution of the phenyl ring with –OCH_3_ did not result in the corresponding cyclometallated complexes. Only *o*-substitution of the phenyl ring with a NH_2_ provided a bimetallic Pt(ii) complex (17) combining cyclometallation and coordination with each of the Pt centres. Additionally, an *in situ* activation of Pt-arylguanidine complexes with acetonitrile to create a bidentate aryl iminoguanidine Pt(ii) complex are reported. The cyclometallation methodology was extended to create a water-stable conjugate incorporating two Pt(ii) ions and a diaryl bis-guanidine DNA minor groove binder previously reported by us. The cyclometallated Pt(ii) complexes were particularly stable to aqueous environments and some of them were tested for ROS generation and anticancer activity in HL-60 cells.

## Data availability

The following ESI† is available: high temperature ^1^H NMR experiments for the formation of complex 15; preparation of mono-guanidinium-like derivatives used as precursors of the ligands used to explore Pt complexation conditions (*i.e.* aryl guanidinium salts 1–7 and the corresponding precursors bis-Boc-protected guanidines S6, S7–S8, S9–S10, S13–S14); experimental details of the methods utilised; reaction of compound 21 with *cis*-[PtCl_2_(DMSO)_2_] followed by ^1^H NMR; graphs showing the experiments with complexes 15 and 16 searching for ROS generation; biochemical details (*i.e.* cell viability assays); X-ray crystallographic data for compounds 15, 16, 17, 18, 19, 20, and 25.

## Author contributions

Conceptualization, I. R. and C. J. M.; methodology, I. R., C. J. M. and A. R. McD.; data analysis, P. O'. S., V. P., B. T., C. J. M., A. R. McD., and I. R.; experiments, P. O'. S., V. P., and B. T.; writing and editing, P. O'. S., V. P., B. T., C. J. M., A. R. McD., and I. R.; supervision, I. R., C. J. M. and A. R. McD. All authors have read and agreed to the published version of the manuscript.

## Conflicts of interest

There are no conflicts to declare.

## Supplementary Material

RA-015-D5RA00310E-s001

RA-015-D5RA00310E-s002
