# Supplementary material for: Platinum(ii) complexes of aryl guanidine-like derivatives as potential anticancer agents: between coordination and cyclometallation
Source: RSC Adv. 2025 Feb 4;15(5):3427–38. doi: 10.1039/d5ra00310e (PMC11791622; doi:10.1039/d5ra00310e)
Supplement: RA-015-D5RA00310E-s001 [file RA-015-D5RA00310E-s001.pdf]

Supplementary Information (SI) for RSC Advances.
This journal is © The Royal Society of Chemistry 2025

Supporting Information

Platinum(II) complexes of aryl guanidine-like derivatives as potential antitumor agents: between coordination and cyclometallation

Patrick O'Sullivan,[a] Viola Previtali,[a] Brendan Twamley,[a] Celine J. Marmion,[b] Aidan R. McDonald,[a] Isabel Rozas[a,*]

[a]*School of Chemistry, Trinity College Dublin, 152-160 Pearse Street, Dublin 2, Ireland*

[b]*Department of Pharmaceutical and Medicinal Chemistry, Royal College of Surgeons in Ireland, 123 St. Stephen's Green, Dublin 2 Ireland*

1. High temperature ^{1}H NMR experiments for the formation of complex 15

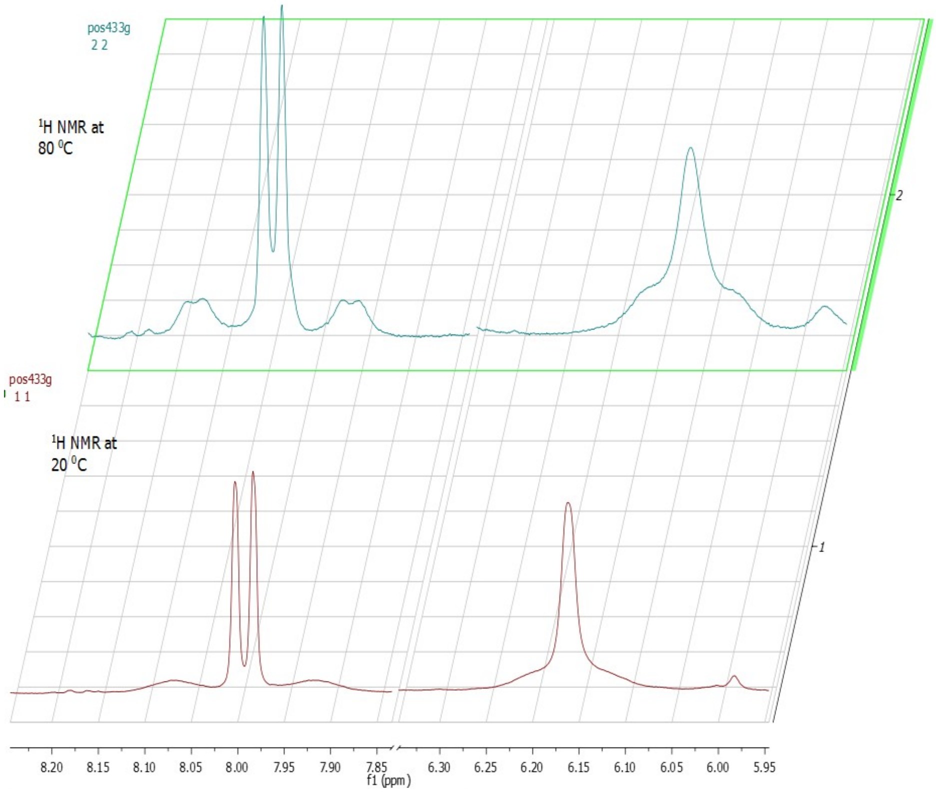

Figure S1. High temperature ^{1}H NMR experiments in DMSO-d_6 on a mixture of **15** clearly demonstrating presence of satellite peaks around the nearest neighbours to Pt. From bottom to top, the temperatures are 20 °C (red) and 80 °C (blue).

2. Synthesis of mono-guanidinium-like derivatives used to optimise Pt complexation conditions.

First, aryl guanidinium salts (**1**,[1] **2**-**3**,[2] **4**-**5**[3]) were prepared *via* the bis-Boc-protected guanidines (**S6**,[4] **S7**-**S8**,[2] **S9**-**S10**[3]) synthesised, as previously reported by our group and others from the reaction of the appropriate aniline/aminopyridine with *N,N'*-bis-(*tert*-butoxycarbonyl)-*S*-methylisothiourea. Subsequent Boc-deprotection was carried out with HCl/dioxane solutions and, in the particular case of Boc-protected *ortho*-amino phenylguanidine **S8**, the alternative use of $SnCl_4$ at room temperature was required. The removal of Sn by-products was easily achieved by trituration and decantation of the crude gum with hexanes until a pure crystalline solid was obtained (Scheme S1). To explore different effects over the guanidinium system, 2-(iminophenyl)imidazolidinium (**6**)[1] and benzylguanidinium (**7**)[5] salts were also prepared from the deprotection of the corresponding Boc-guanidino derivatives (**S13**[1] and **S14**,[2] respectively) (Scheme S1).

Scheme S1. Synthesis of guanidinium-like hydrochlorides **1-7**.

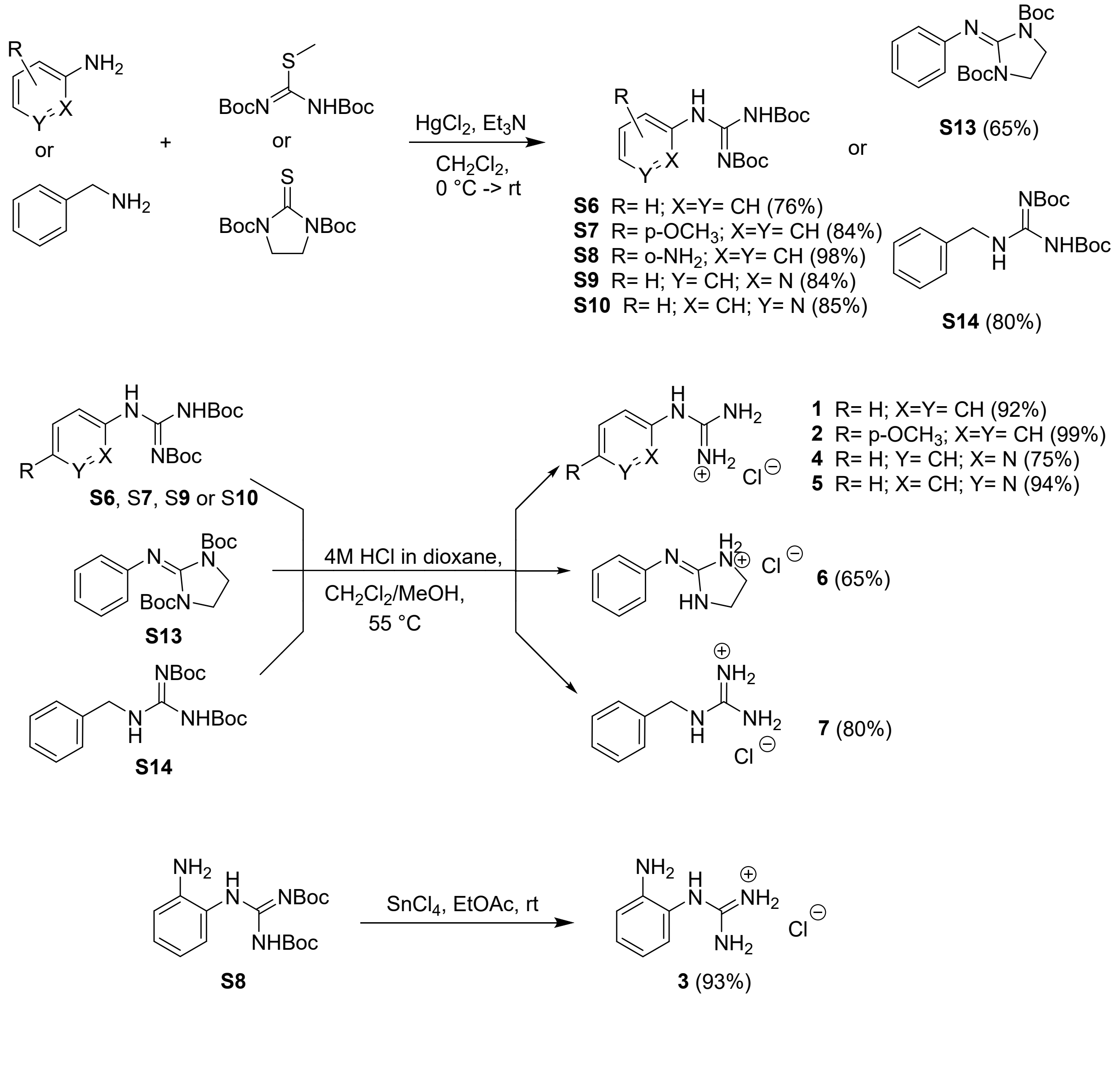

Experimental

All commercial chemicals were obtained from Sigma-Aldrich or Fisher and were used without further purification. Phosphate buffer solutions contained 10 mM K_2HPO_4/KH_2PO_4 adjusted to pH 7 and were prepared using Millipore water. Deuterated solvents for NMR use were purchased from Apollo. Dry DMF, DMSO and 1,4-dioxane were purchased from Sigma-Aldrich or Fisher. THF was dried over 4 Å molecular sieves (activated under vacuum by heating with a heat gun for 30 min) for at least 24 h before use. All other dry solvents were prepared using standard procedures, according to Vogel, with distillation prior to use. Chromatographic columns were run using Silica gel 60 (230-400 mesh ASTM). Solvents for synthetic purposes were used at general purpose reagent (GPR) grade. Analytical TLC was performed using Merck Kieselgel 60 F_{254} silica gel plates. Visualisation was by UV light (254 nm) or by staining with I_2, $KMnO_4$, ninhydrin or cerium ammonium molybdate. NMR spectra were recorded in Bruker DPX-400 Avance spectrometers and operating at 400.13 MHz and 600.1 MHz for ^{1}H-NMR and 100.6 MHz and 150.9 MHz for ^{13}C-NMR and were referenced to the internal solvent signals. For ^{195}Pt-NMR, the spectrometer was operated at 86.04 MHz and was referenced to an external sample of K_2PtCl_4 (-1600 ppm). For ^{19}F-NMR, the spectrometer was operated at 376.20 MHz and was referenced to an external sample of trifluorotoluene (-63.72 ppm). NMR data were processed using MestReNova software. HRMS spectra were measured on a Micromass LCT electrospray TOF instrument with a WATERS 2690 autosampler with methanol as carrier solvent. APCI (atmospheric pressure chemical ionisation) spectra were measured on a micrOTOF-QIII with a direct insertion probe (DIP). Melting points were determined using a Stuart Scientific Melting Point SMP1 apparatus and are uncorrected. Infrared spectra were recorded on a Perkin Elmer Spectrum One FT-IR Spectrometer equipped with a Universal ATR sampling accessory. Microanalysis was performed using an Exeter Analytical CE 440 elemental analyser. HPLC purity analysis was carried out using a Varian ProStar system equipped with a Varian Prostar 335 diode array detector and a manual injector (20 µL). UV detection was performed at 245 nm and peak purity was confirmed using a purity channel. The stationary phase consisted of an ACE 5 C18-AR column (150 x 4.6 mm), and the mobile phase used the following gradient system, eluting at 1.0 cm^3/min: aqueous formate buffer (30 mM, pH 3.0) for ten minutes, linear ramp to 85% methanol buffered with the same system over 25 minutes, held at 85% buffered methanol for ten minutes.

Method A: Synthesis of Boc-protected guanidines.

The appropriate aniline (1.0 eq., 1.47 mmol) was dissolved in dichloromethane or *N*,*N*-dimethylformamide at rt with *N*,*N*-di(*tert*-butoxycarbonyl)-*S*-methyl thiourea, (1.0 eq., 1.47 mmol) and Et_3N (3.1 eq., 4.55

mmol). To this was added $HgCl_2$ (1.1 eq., 1.62 mmol) and the resulting mixture was stirred for 16 h at rt. If the reaction did not go to completion (as adjudged by TLC) a further 0.1 eq. of mercury chloride and Et_3N were added. Upon completion, the reaction mixture was diluted with EtOAc (20 mL) and filtered through a pad of Celite®. The filter cake was rinsed with EtOAc (30 mL) and water (60 mL) was added to the filtrate. The mixture was separated, and the aqueous phase was extracted with EtOAc (2 × 30 mL). The combined organic layer was washed with brine (2 × 30 mL), dried over Na_2SO_4, and concentrated under vacuum. The crude product was purified by silica gel column chromatography, eluting with the appropriate gradient of hexane/ ethyl acetate.

Method B: Boc deprotection reaction.

The appropriate Boc-protected 2-amino-1,4,5,6-tetrahydropyrimidine, 2-aminoimidazoline, guanidine or amine (1.0 eq., 0.47 mmol) was added to a 4 M solution of HCl in dry 1,4-dioxane (6.0 eq. per Boc group, 5.64 mmol) and the solution was brought to 0.2 M concentration by addition of CH_2Cl_2. The solution was stirred at 55 °C until the TLC showed a single highly polar spot (typically 7 h). After 2 h, a small amount of CH_3OH was added if necessary to solubilise the compound. Upon completion, the reaction was then partitioned between water (3 mL) and dichloromethane (3 × 5 mL). The aqueous layer was concentrated under vacuum to give the deprotected compound as a HCl salt.

Method C: Synthesis of guanidine and 2-imino-imidazolidine free bases.

A portion of Na metal (1.2 eq., 0.6 mmol) was cut into small pieces and carefully dissolved in EtOH (0.5 mL) under a constant flow of Ar and stirred for 30 min to give a solution of fresh NaOEt (1.2 eq., 0.6 mmol). This solution was added to a solution of the appropriate guanidinium or 2-amino-imidazolinium salt (1.0 eq., 0.5 mmol) in EtOH (0.5 mL) and stirred for 1.5 h. The mixture was allowed to stand for 30 min and cooled to 0 °C. NaCl was filtered off through filter paper and the filtrate was evaporated under vacuum to give the appropriate free base which was used without further purification.

Method D: Synthesis of cycloplatinated arylguanidine and 2-arylaminoimidazoline complexes.

A suspension of *cis*-[$PtCl_2(dmso)_2$] (1.0 eq., 0.888 mmol), the appropriate free base (1.0 eq., 0.888 mmol) in DMSO (1 mL) or in a fresh NaOMe solution (1.1 eq., 1.00 mmol in MeOH, 24 mL) was heated to 80 °C for 2-48 h. MeOH was removed *in vacuo* and the solid was dissolved in a minimum of DMSO (*ca.* 1 mL), filtered through cotton wool and precipitated using H_2O (5-30 mL). The fine solid was collected by centrifugation (8000 rpm), re-dissolved in DMSO (0.2 mL) and precipitated with H_2O if necessary. The powder was dried at rt *in vacuo* overnight at *ca.* 10 mbar.

_N,N'_-Di-_tert_-butoxycarbonyl-_N''_-phenylguanidine (S6)[5]

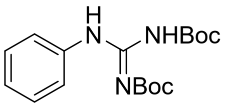

As per Method A, using aniline (0.59 mL, 6.44 mmol), *N,N'*-*bis*(*tert*-butoxycarbonyl)-*S*-methylthiopseudourea (1.963 g, 6.76 mmol), Et_3N (2.78 mL, 20.0 mmol), $HgCl_2$ (1.844 g, 6.78 mmol) and CH_2Cl_2 (15 mL), following purification on silica, eluting in 4% EtOAc in hexane, gave the title compound as a white solid (1.641 g, 76%). **M.p.** 126 °C (lit. 118-120 °C).[5]

δ_H (400 MHz, $CDCl_3$): 1.52 (s, 18H, CH_3), 7.12 (t, 1H, J 7.2, *p*-Ar), 7.30-7.35 (m, 2H, *m*-Ar), 7.57 (d, 2H, J 7.8, *o*-Ar), 10.41 (br s, 1H, NHAr), 11.71 (br s, 1H, NHBoc).

_N,N'_-Di-_tert_-butoxycarbonyl-_N''_-4-methoxyphenylguanidine (S7)[2]

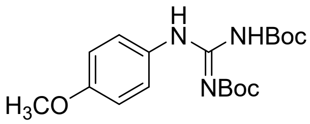

As per Method A, using *p*-anisidine (200 mg, 1.62 mmol), *N,N'*-*bis*(*tert*-butoxycarbonyl)-*S*-methylthiopseudourea (470 mg, 1.62 mmol), Et_3N (0.80 mL, 5.68 mmol), $HgCl_2$ (529 mg, 1.95 mmol) and CH_2Cl_2 (6 mL), following purification on silica, eluting in 5% EtOAc in hexane, gave the title compound as a cream solid (500 mg, 84%). **M.p.** 135-136 °C (lit. 138-140 °C).[2]

δ_H (400 MHz, $CDCl_3$): 1.49 (s, 9H, CH_3 Boc), 1.53 (s, 9H, CH_3 Boc), 3.79 (s, 3H, OCH_3), 6.86 (d, 2H, J 8.9, Ar), 7.48 (d, 2H, J 8.9, Ar), 10.19 (br s, 1H, NHAr), 11.64 (br s, 1H, NHBoc).

_N_-(2-Aminophenyl)-_N',N''_-_bis_(_tert_-butoxycarbonyl)guanidine (S8)[2]

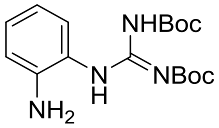

As per Method A, using *o*-phenylenediamine (400 mg, 3.70 mmol), *N,N'*-*bis*(*tert*-butoxycarbonyl)-*S*-methylthiopseudourea (537 mg, 1.85 mmol), Et_3N (0.90 mL, 6.47 mmol), $HgCl_2$ (502 mg, 1.85 mmol) and CH_2Cl_2 (14 mL), following purification on silica, eluting in 10% EtOAc in hexane, gave the title compound as an orange solid (660 mg, 99%). **M.p.** 115 °C.[2]

δ_H (400 MHz, DMSO-d_6): 1.35 (s, 9H, CH_3), 1.51 (s, 9H, CH_3), 5.05 (br s, 2H, NH_2), 6.55 (t, 1H, J 7.6, Ar-5), 6.73 (d, 1H, J 7.1, Ar-3), 6.96 (t, 1H, J 7.0, Ar-4), 7.11 (d, 1H, J 7.6, Ar-6), 9.37 (br s, 1H, NHAr), 11.57 (br s, 1H, NHBoc).

δ_C (100 MHz, DMSO-d_6): 22.7 (CH_3), 22.9 (CH_3), 78.4 (q $C(CH_3)_3$), 83.0 (q $C(CH_3)_3$), 115.6 (CH Ar-3), 116.0 (CH Ar-5), 121.4 (q Ar-2). 127.2 (CH Ar-4), 127.6 (CH Ar-6), 143.5 (q Ar-1), 152.2 (q C=O), 154.9 (q C=O), 163.0 (q C=N).

ν_{max} (ATR)/cm^{-1}: 3407, 3321, 3262, 2980, 1717, 1607, 1406, 1347, 1308, 1147, 1116, 1057, 857, 811, 752.

HRMS (*m/z* ESI$^+$) Found: 373.1854 ($[M+Na]^+$. $C_{17}H_{26}N_4O_4Na$ Requires 373.1852).

***N*-(Pyridin-2-yl)-*N'*,*N"*-*bis*(*tert*-butoxycarbonyl)guanidine (S9)[3]**

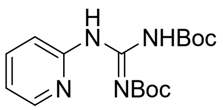

As per Method A, using 2-aminopyridine (200 mg, 2.12 mmol), *N*,*N'*-*bis*(*tert*-butoxycarbonyl)-*S*-methylthiopseudourea (648 mg, 2.23 mmol), Et_3N (1.36 mL, 9.75 mmol), $HgCl_2$ (606 mg, 2.23 mmol) and CH_2Cl_2 (10 mL), following purification on silica, eluting in 4% EtOAc in hexane, gave the title compound as a cream solid (601 mg, 84%). **M.p.** 125 °C (lit. 120-122 °C).[3]

δ_H (400 MHz, $CDCl_3$): 1.51 (br s, 18H, CH_3), 6.97-7.02 (m, 1H, Ar), 7.69 (t, 1H, J 7.9, Ar), 8.28 (d, 1H, J 4.9, Ar), 8.29 (br s, 1H, Ar), 10.91 (br s, 1H, NHAr), 11.48 (br s, 1H, NHBoc).

***N*-(Pyridin-3-yl)-*N'*,*N"*-*bis*(*tert*-butoxycarbonyl)guanidine (S10)[3]**

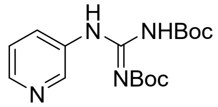

As per Method A, using 3-aminopyridine (200 mg, 2.12 mmol), *N*,*N'*-*bis*(*tert*-butoxycarbonyl)-*S*-methylthiopseudourea (648 mg, 2.23 mmol), Et_3N (1.36 mL, 9.75 mmol), $HgCl_2$ (606 mg, 2.23 mmol) and CH_2Cl_2 (10 mL), following purification on silica, eluting in 4% EtOAc in hexane, gave the title compound as a white solid (607 mg, 85%). **M.p.** 95-96 °C (lit. 99-101 °C).[3]

δ_H (400 MHz, $CDCl_3$): 1.50 (s, 9H, CH_3), 1.54 (s, 9H, CH_3), 7.30 (dd, 1H, J 8.3, J 4.7, Ar), 8.22 (d, 1H, 8.3 Hz, Ar), 8.35 (br s, 1H, Ar), 8.70 (br s, 1H, Ar), 10.42 (br s, 1H, NHAr), 11.60 (br s, 1H, NHBoc).

***N,N'-Bis*(*tert*-butoxy)iminoimidazolidinobenzene (S11)**[1]

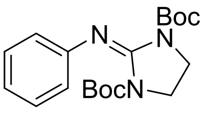

As per *Method A*, using aniline (182 µL, 2.0 mmol), Boc-protected 2-imidazolinethione (605 mg, 2.0 mmol), Et_3N (976 µL, 7.0 mmol), $HgCl_2$ (639 mg, 2.2 mmol) and CH_2Cl_2 (7.5 mL), following recrystallisation from 6:1 hexane/Et_2O, gave the title compound as a white solid (475 mg, 66%). **M.p.** 142-144 °C (lit. 142-144 °C).[1]

δ_H (400 MHz, $CDCl_3$): 1.32 (s, 18H, CH_3), 3.87 (s, 4H, CH_2), 6.97 (t, 1H, J 7.2, *p*-Ar), 7.04 (d, 2H, J 7.2, *o*-Ar), 7.23 (t, 2H, J 7.8, *m*-Ar).

HRMS (*m/z* ESI^+) Found: 151.0975 ($[M+H]^+$. $C_7H_{11}N_4$ Requires 151.0978).

***N*-benzyl-*N'*,*N''*-*bis*(*tert*-butoxycarbonyl)guanidine (S12)**[3]

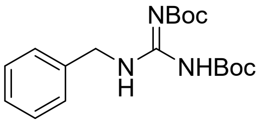

As per Method A, using benzylamine (232 µL, 2.12 mmol), *N*,*N'*-*bis*(*tert*-butoxycarbonyl)-*S*-methylthiopseudourea (648 mg, 2.23 mmol), Et_3N (1.36 mL, 9.75 mmol), $HgCl_2$ (606 mg, 2.23 mmol) and CH_2Cl_2 (10 mL), following purification on silica, eluting in 15% EtOAc in hexane, gave the title compound as a white solid (595 mg, 80%). **M.p.** 123-125 °C. (lit. 124-125 °C).[3]

δ_H (400 MHz, $CDCl_3$): 1.46 (s, 9H, CH_3), 1.50 (s, 9H, CH_3), 4.61 (d, 2H, J 5.1, CH_2), 7.30 (m, 5H, Ar), 8.57 (br s, 1H, NHBn), 11.52 (br s, 1H, NHBoc).

δ_C (100 MHz, $CDCl_3$): 28.0 (CH_3), 28.1 (CH_3), 45.0 (CH_2), 83.2 (q $C(CH_3)$), 127.6 (CH *p*-Ar), 127.8 (CH *o*-Ar), 128.7 (CH *m*-Ar), 137.2 (q Ar), 153.1 (q C=N) 156.1 (q C=O).

Phenylguanidine hydrochloride (1)[2]

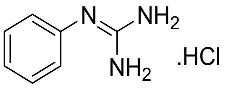

As per Method B, using compound **S6** (1.053 g, 3.14 mmol) and HCl (9.42 mL, 37.7 mmol, 4M in dioxane) in CH_2Cl_2 (6.3 mL), the title compound was obtained as a pale yellow gum (534 mg, 99%).[2]

δ_H (400 MHz, D_2O): 7.28-7.32 (app. d, 2H, *o*-Ar), 7.37-7.42 (app. t, 1H, *p*-Ar), 7.44-7.50 (app. t, 2H, *m*-Ar).

HRMS (*m/z* ESI$^+$) Found: 136.0866 ($[M+H]^+$. $C_7H_{10}N_3$ Requires 136.0869).

4-Methoxyphenylguanidinium hydrochloride (2)[2]

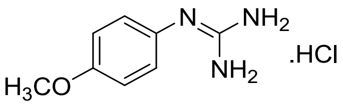

As per Method B using compound **S2** (200 mg, 0.547 mmol) and HCl (1.64 mL, 6.586 mmol, 4M in dioxane) in CH_2Cl_2 (1.1 mL), the title compound was obtained as a white solid (98 mg, 89%). **M.p.** 144-146 °C (lit. 139-141 °C).[2]

δ_H (400 MHz, D_2O): 3.87 (s, 3H, CH_3), 7.09 (d, 2H, J 8.9, Ar), 7.30 (d, 2H, J 8.9, Ar).

HRMS (*m/z* ESI$^+$) Found: 166.0977 ($[M+H]^+$. $C_8H_{12}N_3O$ Requires 166.0975).

2-Aminophenylguanidinium hydrochloride (3)[2]

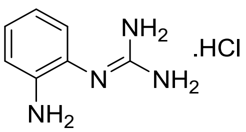

To a solution of **S8** (300 mg, 0.86 mmol) in EtOAc (5 mL) at r.t. was added anhydrous $SnCl_4$ (0.4 mL, 3.42 mmol). The reaction was stirred for 35 min at r.t. and when starting material was consumed (as adjudged by TLC) the solvent was evaporated *in vacuo* and then any remaining $SnCl_4$ was quenched with MeOH (1 mL). Upon rotary evaporation of MeOH, the crude oil was dissolved in hexane to give a bilayer from which the hexane layer was decanted. This procedure was repeated until the title compound was precipitated as a dark purple solid (150 mg, 93%). **M.p.** 244 °C.

δ_H (400 MHz, DMSO-d_6): 5.19 (br s, 2H, NH_2),6.59 (t, 1H, J 7.4, Ar-5), 6.79 (d, 1H, J 7.9, Ar-3),6.96 (d, 1H, J 7.4, Ar-6), 7.03 (br s, 4H, NH_2), 7.04-7.09 (m, 1H, Ar-4), 8.81 (br s, 1H, NH).

δ_C (100 MHz, DMSO-d_6): 115.9 (CH Ar-3), 116.5 (CH Ar-5), 118.6 (q Ar-2), 128.2 (CH Ar-6), 128.9 (CH Ar-4), 145.1 (q Ar-1), 156.5 (q C=N).

ν_{max} (ATR)/cm^{-1}: 3411 (NH), 3311 (NH), 3159 (NH), 1674, 1654, 1590, 1493, 1269, 1123, 759.

HRMS (*m/z* ESI$^+$) Found: 151.0976 ($[M+H]^+$. $C_7H_{11}N_4$ Requires 151.0978).

2-(Phenylamino)imidazolinium hydrochloride (6)[2]

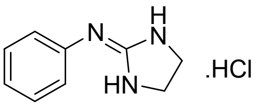

As per Method B using compound **S11** (475 mg, 1.31 mmol) and HCl (3.9 mL, 15.8 mmol, 4M in dioxane) in CH_2Cl_2 (2.6 mL), the title compound was obtained as a white solid (254 mg, 98%). **M.p.** 214-216 °C (lit. 211-213 °C).[2]

δ_H (400 MHz, D_2O): 3.79 (s, 4H, CH_2), 7.32 (d, 2H, J 7.5), 7.40 (t, 1H, J 7.5, Ar), 7.51 (app. t, 2H, J 7.8, Ar).

HRMS (*m/z* ESI$^+$) Found: 162.1022 ($[M+H]^+$. $C_9H_{12}N_3$ Requires 162.1026).

Benzylguanidinium hydrochloride (7)[3]

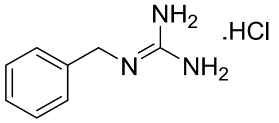

As per Method B using compound **S12** (581 mg, 1.66 mmol) and HCl (4.99 mL, 19.9 mmol, 4M in dioxane) compound **7** was obtained as a yellow gum (121 mg, 79%).

δ_H (400 MHz, D_2O): 4.23 (s, 2H, CH_2), 7.15-7.21 (m, 3H, *o*-Ar and *p*-Ar), 7.22-7.29 (m, 2H, *m*-Ar).

HRMS (*m/z* ESI$^+$) Found: 150.1028 ($[M+H]^+$. $C_8H_{12}N_3$ Requires 150.1031).

Phenylguanidine (8)

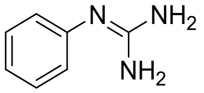

As per Method C, using guanidinium chloride salt **1** (562 mg, 3.27 mmol) and Na metal (83 mg, 3.6 mmol) in EtOH (6.4 mL), afforded the title compound as a yellow solid (439 mg, 99%). **M.p.** 139 °C

δ_H (400 MHz, DMSO-d_6): 6.84 (d, 2H, J 7.5, *o*-Ar), 6.88 (t, 1H, 7.8, *p*-Ar), 7.18-7.24 (app. t, 2H, *m*-Ar).

δ_C (100 MHz, DMSO-d_6): 123.1 (CH p-Ar), 123.9 (CH *o*-Ar), 129.6 (CH *m*-Ar), 144.7 (q Ar), 154.3 (q C=N).

ν_{max} (ATR)/cm^{-1}: 3434 (NH), 3377 (NH), 3316 (NH), 3118, 2984, 2758, 1678, 1624, 1577, 1477, 1343, 1269, 876, 754, 688.

HRMS (*m/z* ESI$^+$) Found: 136.0876 ($[M+H]^+$. $C_7H_{10}N_3$ Requires 136.0875).

4-Methoxyphenylguanidine (9)

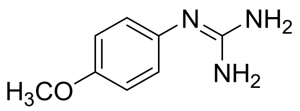

As per Method C, using guanidinium chloride salt **2** (800 mg, 3.97 mmol) and Na metal (100 mg, 4.36 mmol) in EtOH (22 mL), afforded the title compound as a yellow solid (684 mg, 104% including NaOEt). **M.p.** 205 °C.

δ_H (400 MHz, DMSO-d_6): 3.67 (s, 3H, CH_3), 4.30 (br s, 4H, NH_2), 6.70 (d, 2H, J ,Ar-2), 6.77 (d, 2H, J , Ar-3).

δ_C (100 MHz, DMSO-d_6): 55.1 (CH_3), 114.3 (CH Ar-3), 123.7 (CH Ar-2), 142.8 (q Ar-4), 152.7 (q C=N), 153.7 (q Ar-1).

ν_{max} (ATR)/cm^{-1}: 3473 (NH), 3314 (NH), 3171, 1648, 1554, 1491, 1438, 1232, 1232, 1101, 853 811.

HRMS (*m/z* ESI$^+$) Found: 166.0971 ([M+H]$^+$. $C_8H_{12}N_3O$ Requires 166.0975).

2-Aminophenylguanidine (10)

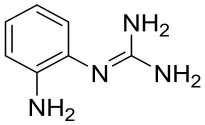

As per Method C, using guanidinium chloride salt **3** (100 mg, 0.54 mmol) and Na metal (13.5 mg, 0.59 mmol) in EtOH (5 mL), afforded the title compound as a yellow solid (85 mg, 105% including NaOEt). **M.p.** 260 °C.

δ_H (400 MHz, DMSO-d_6): 4.78 (br s, 2H, NH_2),6.19 (br s, 4H, NH_2), 6.51 (t, 1H, J 7.5, Ar-C5), 6.67 (d, 1H, J 8.0, Ar-C3),6.77 (d, 1H, J 7.7, Ar-C6), 6.82, (d, 1H, J 7.1, Ar-C4).

δ_C (100 MHz, DMSO-d_6): 115.9 (CH Ar-3), 116.5 (CH Ar-5), 118.6 (q Ar-1), 128.2 (CH Ar-6), 128.9 (CH Ar-4), 145.1 (q Ar-2), 156.5 (q C=N).

ν_{max} (ATR)/cm^{-1}: 3320 (NH), 3145 (NH), 1674, 1637, 1584, 1499, 1269, 836, 757.

HRMS (*m/z* ESI$^+$) Found: 151.0977 ([M+H]$^+$. $C_7H_{11}N_4$ Requires 151.0978).

Pyridine-2-guanidine (11)

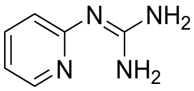

As per Method C, using guanidinium chloride salt **4** (155 mg, 0.90 mmol) and Na metal (25 mg, 1.08 mmol) in EtOH (4 mL), afforded the title compound as an orange solid (133 mg, 108% including NaOEt). **M.p.** 134-136 °C.

δ_H (600 MHz, DMSO-d_6): 6.59-6.62 (m, 2H, H-4 and H-5), 6.74 (br s, 4H, NH), 7.43 (br s, 1H, H-3), 8.04 (br s, 1H, H-6).

δ_C (150 MHz, DMSO-d_6): 113.5 (CH Ar-5), 118.8 (CH Ar-4), 136.3 (CH Ar-3), 145.8 (CH Ar-6), 157.5 (q C=N), 163.7 (q Ar-2).

ν_{max} (ATR)/cm^{-1}: 3202 (NH), 3129 (NH), 2953 (CH), 2765, 1702, 1638, 1603, 1554, 1472, 1419, 1383, 1324, 1284, 1144, 875, 769, 672.

HRMS (*m/z* ESI$^+$) Found: 137.0822 ([M+H]$^+$. $C_6H_9N_4$ Requires 137.0827).

Pyridine-3-guanidine (12)

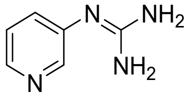

As per Method C, using guanidinium chloride salt **5** (250 mg, 1.45 mmol) and Na metal (37 mg, 1.59 mmol) in EtOH (8 mL) and H_2O (0.5 mL), afforded the title compound as a white solid (220 mg, 110% including NaOEt). **M.p.** 148-149 °C.

δ_H (600 MHz, DMSO-d_6): 7.43 (dd, 1H, J 8.1, J 4.8, H-4), 7.62 (ddd, 1H, J 8.1, J 2.5, J 1.6, H-5), 7.73 (br s, 4H, NH_2), 8.41-8.43 (m, 2H, H-2 and H-6).

δ_C (150 MHz, DMSO-d_6): 124.2 (CH Ar-5), 131.9 (CH Ar-4), 134.0 (q Ar-3), 145.7 (CH Ar-2), 146.5 (CH Ar-6), 156.1 (q C=N).

ν_{max} (ATR)/cm^{-1}: 3362 (NH), 3297 (NH), 3209, 2980 (CH), 2938, 1678, 1635, 1592, 1483, 1399, 1326, 1269, 1192, 1162, 1030, 856, 806.

HRMS (*m/z* ESI$^+$) Found: 137.0819 ([M+H]$^+$. $C_6H_9N_4$ Requires 137.0822).

Imidazolidine-2-phenylimine (13)

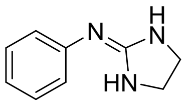

As per Method C, using 2-aminoimidazolinium hydrochloride salt **6** (100 mg, 0.51 mmol) and Na metal (15 mg, 0.61 mmol) in EtOH (1 mL), afforded the title compound as a white solid (76 mg, 93%). **M.p.** 144-145 °C.

δ_H (400 MHz, DMSO-d_6): 3.52 (s, 4H, CH_2), 6.10 (br s, 2H, NH), 7.08 (t, 1H, *p*-Ar), 7.18 (d, 2H, *o*-Ar), 7.32 (t, 2H, *m*-Ar).

δ_C (100 MHz, DMSO-d_6): 43.9 (CH_2), 121.3 (CH *p*- Ar), 122.0 (CH *o*-Ar), 129.5 (CH *m*-Ar), 146.7 (q Ar), 158.2 (q C=N).

ν_{max} (ATR)/cm^{-1}: 3358, 3312 (NH), 3053 (NH), 2860 (CH), 1654, 1587, 1482, 1416, 1266, 1245, 1086, 835, 775, 691.

HRMS (*m/z* ESI$^+$) Found: 162.1027 ($[M+H]^+$. $C_9H_{12}N_3$ Requires 162.1022).

Benzylguanidine (14)

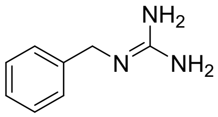

As per Method C, using guanidinium chloride salt **7** (94 mg, 0.50 mmol) and Na metal (13 mg, 0.55 mmol) in EtOH (5 mL), afforded the title compound as an orange solid (78 mg, 104% including NaOEt). **M.p.** <300 °C.

δ_H (400 MHz, DMSO-d_6): 4.09 (s, 2H, CH_2), 7.23-7.28 (m, 3H, *o*-Ar & *p*-Ar), 7.36-39 (m, 2H, *m*-Ar).

δ_C (150 MHz, DMSO-d_6): 44.3 (CH_2), 127.2 (CH *o*-Ar & *p*-Ar), 128.4 (CH *m*-Ar), 138.2 (q Ar), 157.1 (C=N).

ν_{max} (ATR)/cm^{-1}: 3240 (NH), 3137 (NH), 2984 (CH), 1637, 1453, 1349, 752.

HRMS (*m/z* ESI$^+$) Found: 150.1033 ($[M+H]^+$. $C_8H_{12}N_3$ Requires 150.1031).

4-4'-Bis-guanidinobenzophenone (21)

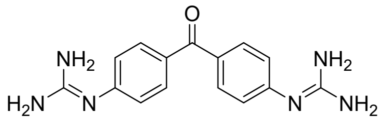

As per Method C, using the corresponding bis-guanidinium salt prepared as per reference 1 (219 mg, 0.59 mmol) and Na metal (30 mg, 1.31 mmol) in EtOH (2.5 mL), afforded the title compound as a white gum (178 mg, 102% with NaOEt).

δ_H (400 MHz, DMSO-d_6): 5.61 (br s, 8H, NH_2), 6.91 (d, 4H, J 8.5, Ar-2), 7.59 (d, 4H, J 8.5, Ar-3).

δ_C (100 MHz, DMSO-d_6): 127.3 (Ar CH-2), 134.4 (q Ar-1), 136.1 (Ar CH-3), 158.5 (q Ar-4), 159.7 (q C=N), 198.3 (q C=O).

ν_{max} (ATR)/cm^{-1}: 3362 (NH), 3312 (NH), 3066, 1630 (C=N), 1560, 1532, 1441, 1406, 1309, 1170, 930, 864, 771.

HRMS (*m/z* ESI$^+$) Found: 297.1460 ($[M+H]^+$. $C_{15}H_{17}N_6O$ Requires 297.1464).

Di-(4-guanidinophenyl)ether (22)

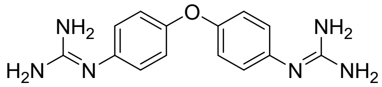

As per Method C, using the corresponding bis-guanidinium salt prepared as per reference 1 (420 mg, 1.18 mmol) and Na metal (51 mg, 2.60 mmol) in EtOH (6.0 mL), afforded the title compound as a white solid (356 mg, 99%). **M.p.** 205-207 °C.

δ_H (400 MHz, DMSO-d_6): 5.18 (br s, 8H, NH), 6.72 (d, 4H, 8.8 Hz, Ar), 6.80 (d, 4H, 8.8 Hz, Ar).

δ_C (100 MHz, DMSO-d_6): 119.3 (CH Ar-3), 124.3 (CH Ar-2), 146.3 (q Ar-1), 151.5 (q C=N), 153.0 (q Ar-4).

ν_{max} (ATR)/cm^{-1}: 3457 (NH), 3332 (NH), 3179, 3031, 2930 (CH), 1635, 1583, 1489, 1436, 1219, 855, 810.

HRMS (*m/z* ESI$^+$) Found: 285.1461 ($[M+H]^+$. $C_{14}H_{17}N_6O$ Requires 285.1464).

a-Chlorido-b-(dmso-S)-cd-(2-phenylyl-κC²-guanidine-κN)platinum(II) (15)

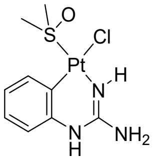

As per Method D, free base **8** (119 mg, 0.888 mmol) and *cis*-[$PtCl_2(dmso)_2$] (360 mg, 0.888 mmol) were added to a freshly prepared NaOMe solution produced from Na metal (24 mg, 1.00 mmol) and MeOH (24 mL), affording after precipitation the title compound as a yellow solid (94 mg, 24%). Crystals suitable for XRD were grown over three months by slow evaporation of H_2O into a concentrated solution of the title compound in DMSO.

δ_H (400 MHz, DMSO-d_6): 3.35 (s, 6H, CH_3), 6.19 (br s, 1H, PtNH), 6.31 (br s, 2H, NH_2), 6.59-6.72 (m, 2H, Ar-3 & Ar-4), 6.96 (t, 1H, J 7.4, Ar-5), 7.97 (d+dd, 1H, J 7.7 + J 62.6, Ar-6), 9.13 (br s, 1H, ArNH).

δ_C (100 MHz, DMSO-d_6): 46.2 (CH_3), 113.1 (q Ar-1), 115.0 (CH Ar-3), 121.2 (CH Ar-4), 124.1 (CH Ar-5), 137.0 (q Ar-2), 138.3 (CH Ar-6), 150.4 (q C=N).

δ_{Pt} (86 MHz, DMSO-d_6): -3582.

ν_{max} (ATR)/cm^{-1}: 3407 (NH), 3310 (N), 3193 (NH), 2352, 1645 (C=N), 1602, 1551, 1479, 1399, 1300, 1091 (S-O), 1022, 758, 721.

% Calculated for $C_9H_{14}N_3OPtClS\cdot 0.5NaCl\cdot 1.5H_2O\cdot 0.5DMSO$: C 22.32, H 3.28, N 7.81. **% Found:** C 22.12, H 3.01, N 8.01.

LRMS (*m/z* ESI$^+$) Found: 444.2 ($[M+H]^+$. $C_9H_{15}N_3OSCl_2Pt$ Requires 444.03).

a-Chlorido-b-(dmso-S)-cd-([2-aminophenylyl]-κC²-imidazoline-κN)platinum(II) (16)

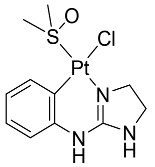

As per Method D, free base **13** (200 mg, 1.15 mmol) and *cis*-[$PtCl_2(dmso)_2$] (462 mg, 1.15 mmol) were added to a freshly prepared NaOMe solution produced from Na metal (29 mg, 1.18 mmol) and MeOH (25 mL), affording after precipitation the title compound as a dark blue solid (90 mg, 18%). **M.p.** 223-224 °C. Crystals suitable for XRD were grown over three months by slow evaporation of H_2O into a concentrated solution of the title compound in DMSO.

δ_H (400 MHz, DMSO-d_6): 3.14-3.57 (m, 8H, 2 x CH_3 & CH_2), 3.99 (br s, 2H, CH_2), 6.37-6.77 (m, 2H, Ar-3 & Ar-4), 6.77-7.11 (m, 1H, Ar-5), 7.11-7.57 (m, 1H, NH), 7.57-8.01 (m, 1H, Ar-6), 9.53 (br s, 1H, NH).

δ_C (100 MHz, DMSO-d_6): 44.0 (CH_2), 46.1 (CH_3), 51.5 (CH_2), 114.9 (q Ar-1), 115.0 (CH Ar-4), 121.8 (CH Ar-3), 124.6 (CH Ar-5), 137.8 (q Ar-2), 139.6 (CH Ar-6), 156.0 (q C=N).

δ_{Pt} (86 MHz, DMSO-d_6) -3633.

ν_{max} (ATR)/cm^{-1}: 3339, 3282 (NH), 3185 (NH), 2994 (CH), 1606, 1578, 1465, 1413, 1288, 1094, 1017, 743.

% Calculated for $C_{11}H_{17}N_3OPtClS \cdot H_2O \cdot 0.5DMSO$: C 27.35, H 3.73, N 7.97. **% Found:** C 27.16, H 3.45, N 8.16.

Bis-Pt Complex of 2-aminophenylguanidine (17)

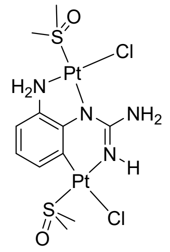

As per Method D, free base **10** (15 mg, 0.1 mmol) and *cis*-[$PtCl_2$(dmso)$_2$] (20 mg, 0.05 mmol) were dissolved in DMSO-d_6 (1 mL), affording after precipitation the title compound as a dark purple solid (6 mg, 24%). Crystals suitable for XRD were grown over three months by slow evaporation of H_2O into a concentrated solution of the title compound in DMSO.

Triiodo-*tris*(4-methoxyguanidine)*bis*-platinum (III) complex (18)

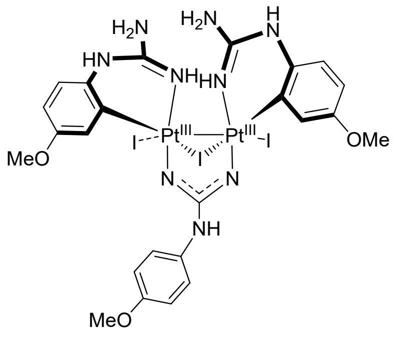

To K_2PtCl_4 (41 mg, 0.1 mmol) in H_2O (1 mL) at 60 °C, was added KI (100 mg, 0.6 mmol). The mixture was stirred in the dark for 20 min, after which free base **9** (37 mg, 0.2 mmol) was added. The yellow mixture was stirred at r.t. for 15 min and the brown precipitate was filtered and washed with H_2O. The

compound was dissolved in EtOAc and crystallised out by slow evaporation of hexane to give crystals suitable for XRD.

No further characterisation data is available for this compound.

trans-[Dichloro(dmso)(2-[phenylamino]imidazoline-κ*N*)platinum] (19)

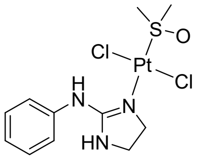

To a solution of K_2PtCl_4 (139 mg, 0.335 mmol) in H_2O (0.5 mL) was added dropwise a solution of DMSO (24 µL, 0.335 mmol) in H_2O (0.5 mL) and the reaction was stirred at r.t. for 4 h until the colour changed from red to yellow. The solid free base **13** (54 mg, 0.335 mmol) and CH_2Cl_2 (0.5 mL) were then added and the reaction was stirred at r.t. for 16 h. The organic layer was separated and evaporated at r.t. by blowing with Ar to give a crude oil which was purified on silica, eluting in 0.5% acetone in CH_2Cl_2 to give the title compound as a yellow solid (33 mg, 19%). Crystals suitable for XRD were grown over 4 h by slow evaporation of Et_2O into a concentrated solution of the title compound in CH_2Cl_2.

δ_H (400 MHz, DMF-*d*$_7$): 3.37 (s, 6H, CH_3), 3.59 (t, 2H, J 9.1, CH_2), 3.85 (t, 2H, J 9.1, CH_2), 7.15 (br s, 1H, NH), 7.20 (t, 1H, J 7.4, *p*-Ar), 7.30 (d, 2H, J 7.5, *o*-Ar), 7.38-7.43 (m, 2H, *m*-Ar), 8.88 (br s, 1H, NH).

δ_C (100 MHz, DMF-*d*$_7$): 42.5 (CH_3), 43.5 (CH_2), 51.4 (CH_2), 123.1 (CH *o*-Ar), 124.8 (CH *p*-Ar), 129.4 (CH *m*-Ar), 138.6 (q Ar), 160.7 (q C=N).

δ_{Pt} (86 MHz, DMF-*d*$_7$): -3033.

ν_{max} (ATR)/cm^{-1}: 3289 (NH), 3090 (NH), 1617 (C=N), 1093 (S-O), 3018, 1588, 1571, 1478, 1432, 1267, 1022, 730 .

HRMS (*m/z* ESI$^-$) Found: 503.0063 ([M-H]$^-$. $C_{11}H_{16}N_3OSCl_2Pt$ Requires 503.0039).

cis-[Dichloro(phenyliminoguanidine (*N,N'*))platinum] (20)

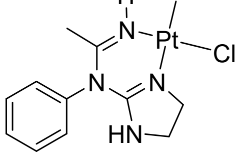

To a crude mixture of **19** prior to column chromatography was added CH_3CN (1 mL). Crystals suitable for XRD were grown over a period of months from this standing solution.

δ_H (400 MHz, DMF-*d₇*): 3.52-3.58 (m, 2H, CH_2), 4.25-4.33 (m, 2H, CH_2), 6.55 (br s 1H, NH), 7.75-7.81 (m, 3H, Ar), 7.86-7.90 (m, 2H, Ar), 10.04 (br s, 1H, NH). NOTE: CH_3 obscured by H_2O peak.

δ_C (100 MHz, DMF-d_7): decomposed over time of experiment (v dilute).

ν_{max} (ATR)/cm^{-1}: 3390, 3254 (NH), 2350, 2164 (CN), 1622(C=N), 1576, 1436, 1271, 1098, 1030, 730.

4,4'-Bis-[*a*-Chlorido-*b*-(dmso-*S)*-*cd*-(2-phenylyl-*κC*^{2}-guanidine-*κN*)platinum(II)]ketone (25)

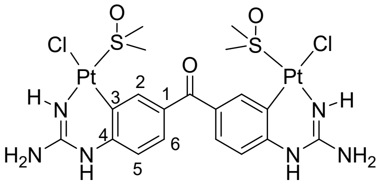

As per Method D, free base **21** (15 mg, 0.05 mmol) and *cis*-[$PtCl_2(dmso)_2$] (40.5 mg, 0.1 mmol) dissolved in DMSO-d_6 (1 mL) at 80 °C for 48 h, afforded after precipitation the title compound as a yellow solid (16 mg, 32%). **M.p.** 250 °C. Crystals suitable for XRD were grown over three months by slow evaporation of H_2O into a concentrated solution of the title compound in DMSO.

δ_H (400 MHz, DMSO-d_6): 3.36 (s, 12H, CH_3), 6.40 (d, 2H, J 2.5, PtNH), 6.43 (s, 4H, NH_2), 6.75 (d, 2H, J 8.2, Ar-5), 7.38 (dd, 2H, J 1.9, J 8.2, Ar-6), 8.48 (d, 2H, J 1.9, Ar-2), 9.48 (d, 2H, J 2.5, NH).

δ_C (150 MHz, DMSO-d_6): 46.0 (CH_3), 112.2 (q Ar C-3), 114.4 (Ar C-5), 126.4 (Ar C-6), 131.0 (Ar C-1), 140.3 (q Ar C-4), 140.8 (Ar C-2), 150.1 (q C=N), 194.4 (q C=O).

δ_{Pt} (86 MHz, DMSO-d_6): -3556, -3559.

ν_{max} (ATR)/cm^{-1}: 3356 (NH), 3206 (NH), 3000, 2917 (CH), 1649, 1619, 1535, 1477, 1304, 1288, 1249, 1093, 1017, 1002, 947, 823, 748, 677.

% Calculated for $C_{19}H_{26}N_6O_3Pt_2Cl_2S_2 \cdot 6NaCl \cdot 6H_2O \cdot 4DMSO$: C 19.27, H 2.55, N 4.99. **% Found:** C 19.32, H 2.90, N 5.21.

3. Reaction of 21 (X = CO) with cis-[$PtCl_2(DMSO)_2$] followed by ^1H NMR

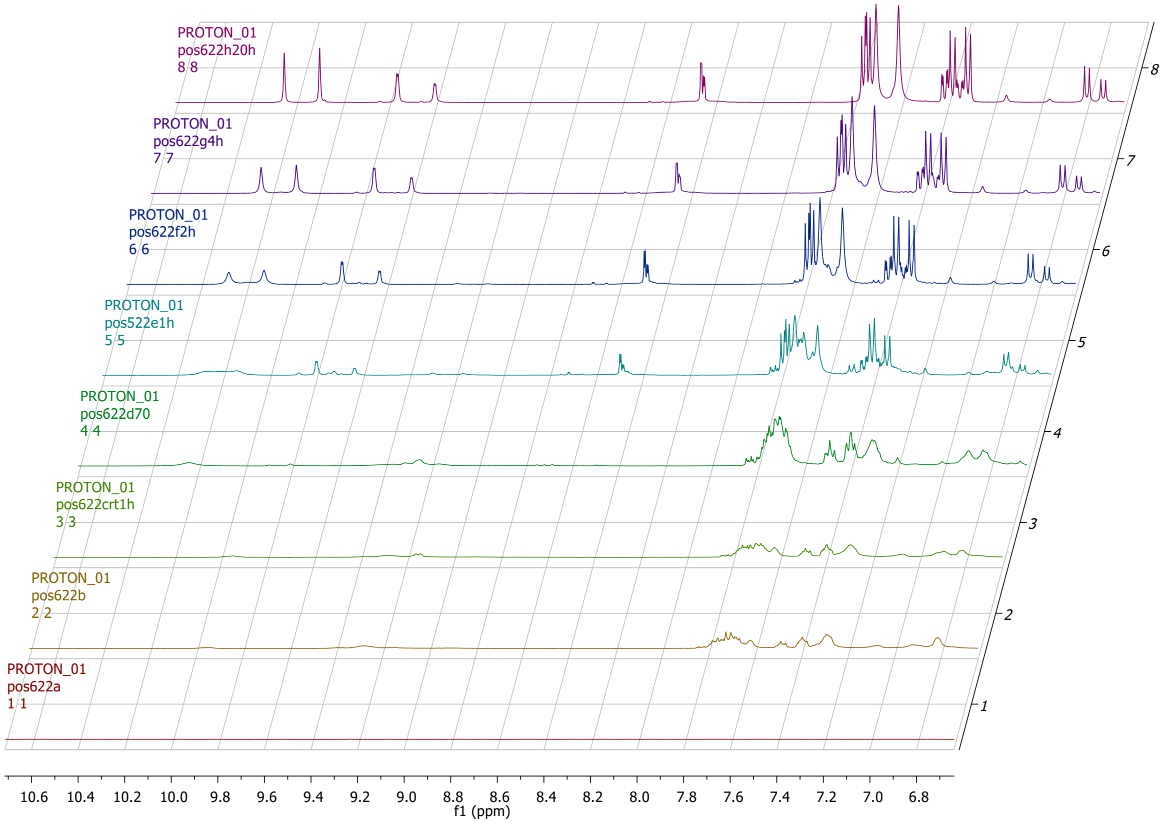

Figure S2. Overlay of ^1H NMR spectra from reaction of 21 (X = CO) with cis-[$PtCl_2(DMSO)_2$].

4. Generation of ROS species

The $^1O_2$ trap compound 1,3-diphenyl-2-benzofuran (DPBF, λ_{max} = 418 nm in DMSO) was used to measure how much $^1O_2$ may be produced when there is a decrease in absorbance at 418 nm. The corresponding complex solution in DMSO (**15** or **16**) was added to the cuvette, and its absorbance was adjusted to around 0.01 at the wavelength of irradiation. The solutions in the cuvette were irradiated with white light and the absorbance of DPBF measured. Absorption spectra were recorded using a Shimadzu UV-1601PC spectrophotometer. Measurements were performed with 10 mm quartz cuvettes (Hellma Precision) with a Teflon cap allowing for gas-purging using syringe needles. A Hamamatsu R928 PMT was used for detection in the range 185–950 nm. A OB-75X (75 W Xenon arc lamp) was used as the light source.

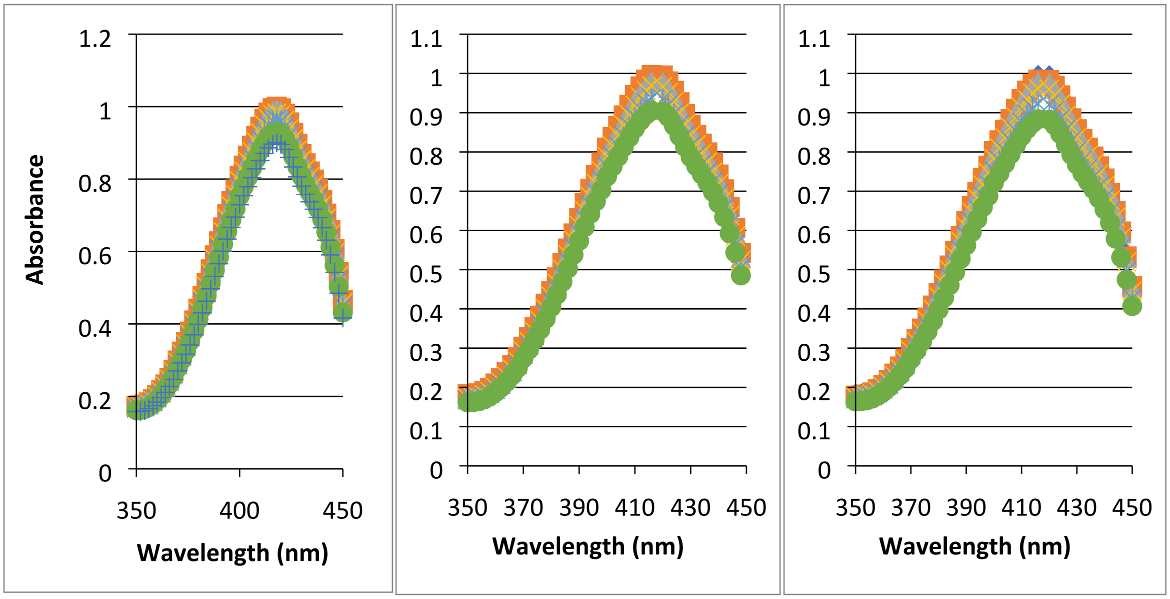

(a) **(b)** **(c)**

Figure S3. Graph showing decrease in concentration of DPBF in the presence of white light and (a) DMSO only, (b) **15** in DMSO, (c) **16** in DMSO at time points of 0, 2, 5, 10, 15, 20, 30 min.

5. Biochemical details

The HL-60 (human caucasian promyelocytic leukemia) cell line was obtained from European Collection of Cell Cultures (Porton Down, Wiltshire, U.K.) and it was maintained between 200,000 – 2,000,000 cells/mL in Roswell Park Memorial Institute (RPMI) 1640 medium with stable glutamate (GlutaMax I) supplemented with 10% (v/v) foetal bovine serum (FBS) and 50 µg/mL penicillin/streptomycin (pen-strep). The growth medium was stored in the fridge at 4 °C and heated to 37 °C prior to culture work. Cells were grown at 37 °C in a humidified environment maintained at 95% O_2 and 5% CO_2 and passaged at least three times weekly depending on their levels of confluency. When required for sub-culturing, cells were transferred to a sterile tube and centrifuged at 1296 rpm for 5 min. The supernatant was discarded, and the cell pellet was resuspended in fresh medium. Cells were then counted using a haemocytometer slide and seeded at the required density.

Cell Viability Assays

HL-60 cells in the log phase of growth were seeded in 96-well plates at a density of 50,000 cells/mL (200 µL/well or 10,000 cells/well) or 200,000 cells/mL (200 µL/well or 40,000 cells/well) in complete RPMI medium the same day of the experiment. Compounds **15**, **16**, **17** and **25** were dissolved in DMSO to obtain a starting 100 mM stock solution. The cells were then treated with either 2 µL of a 1:100 dilution of stock concentrations of drugs or ddH_2O as vehicle control, or 0.2 µL of a 1:1000 dilution of stock concentrations of drugs or DMSO as vehicle control. All experiment were repeated in triplicate for at least three times. Three wells containing 200 µL RPMI with no cells were also set up as blanks.

After 72 h incubation, 20 µL AlamarBlue® was added to each well. The plates were incubated in darkness at 37 °C for 5 hours. Using a Molecular Devices microplate reader, the fluorescence (F) was then read at an excitation wavelength of 544 nm and an emission wavelength of 590 nm. Cell viability was then determined by subtracting the mean blank fluorescence (Fb) from the treated sample fluorescence (Fs) and expressing this as a percentage of the fluorescence of the blanked vehicle control (Fc). This is demonstrated in the equation below. The results were then plotted as nonlinear regression, sigmoidal dose-response curves on Prism GraphPad 5 software, from which the IC_{50} value for each drug was determined.

$$\frac{(Fs - Fb)}{(Fc - Fb)} \times \frac{100}{1} = \%\ Cell\ Viability$$

6. X-ray Crystallography Data

Crystals were mounted on a MiTeGen micromount with NVH immersion oil. Data were collected from a shock-cooled single crystal at 100.00 K on a Bruker Apex Kappa Duo Kappa diffractometer with a standard sealed X-ray tube using a graphite monochromator and a CCD area detector. The diffractometer was equipped with an Oxford Cobra low temperature device and used MoK_{α} radiation (λ = 0.71073 Å). All data were integrated with SAINT and a multi-scan absorption correction using SADABS was applied.[6,7] The structure was solved by dual methods using SHELXT and refined by full-matrix least-squares methods against F^2 by SHELXL using Olex2.[8,9,10] All non-hydrogen atoms were refined with anisotropic displacement parameters. All C-bound hydrogen atoms were refined isotropic on calculated positions using a riding model with their U_{iso} values constrained to 1.5 times the U_{eq} of their pivot atoms for terminal sp^3 carbon atoms and 1.2 times for all other carbon atoms. Disordered moieties were refined using bond lengths restraints and displacement parameter restraints.

In **17**, problems with the crystal alignment during measurement as well as a good amount of diffuse contribution of the sample led to a worse result in the absorption correction. The absorption correction was manually adjusted until the optimum was obtained. However, it is a reasonable assumption that the remaining large residuals are an artefact of the absorption correction due to these two issues. The N4 hydrogens were refined semi-free with restraints (DFIX, DANG).

In the structure of **18**, C24/O23 and C41 were modelled in two locations (84:16% and 46:54% occupied) using geometric and displacement restraints and constraints (SADI, RIGU, EADP). O23A and O23b were modelled using constraints (EXYZ and EADP). Donor N-H hydrogens were located on the difference map and then refined with a riding model (AFIX).

In **20**, N-H hydrogen atoms were located and refined using a DFIX restraint.

In **25(i)**, one Pt-Cl moiety (Pt2-Cl2) was disordered and modelled over two locations (72:28% occupancy). The bridge C=O was disordered over two locations (74:26%). Water hydrogens were placed to optimise hydrogen bond contacts and restrained (AFIX). One water O5s was modelled with the same occupancy as Cl2a (72%). Disordered moieties were modelled using restraints (RIGU, SIMU, SADI, DFIX) and constraints (EADP, EXYZ). The structure was modelled as an inversion twin. The high residual (2.8 e/A3) is ca 0.9 Angstroms from the Pt1 centre and is an absorption artefact.

In **25(ii)**, the water hydrogens were located on the difference map and refined with a riding model (AFIX).

Crystallographic data for the structures reported here have been deposited with the Cambridge Crystallographic Data Centre.[11] CCDC 2391910-2391917 contain the supplementary crystallographic data for this paper. These data can be obtained free of charge from The Cambridge Crystallographic Data Centre via www.ccdc.cam.ac.uk/structures. This report was generated using FinalCif.[12]

Table S1. Structure Tables

Code	**15**	**16**	**17**	**18**	**19**	**20**	**25(i)**	**25(ii)**
Identification	tcd152	TCD177	tcd259	tcd230	tcd181	tcd193	tcd257	TCD309
CCDC number	2391910	2391911	2391912	2391913	2391914	2391915	2391916	2391917
Empirical formula	$C_9H_{14}ClN_3OPtS$	$C_{11}H_{16}ClN_3OPtS$	$C_{11}H_{20}Cl_2N_4O_2Pt_2S_2$	$C_{28}H_{44}I_3N_9O_6Pt_2$	$C_{11}H_{17}Cl_2N_3OPtS$	$C_{13}H_{17}Cl_2N_5Pt$	$C_{19}H_{35.46}Cl_2N_6O_{7.73}Pt_2S_2$	$C_{19}H_{30}Cl_2N_6O_5Pt_2S_2$
Formula weight	442.83	468.87	765.51	1373.60	505.32	509.30	996.83	947.69
Temperature [K]	100(2)	100(2)	100(2)	100(2)	100(2)	100(2)	100(2)	100(2)
Crystal system	orthorhombic	Orthorhombic	monoclinic	Monoclinic	orthorhombic	monoclinic	orthorhombic	Triclinic
Space group (number)	$Pna2_1$ (33)	$Pbca$ (61)	$P2_1/n$ (14)	$P2_1/c$ (14)	$P2_12_12_1$ (19)	$P2_1/c$ (14)	$Pna2_1$ (33)	$P\overline{1}$ (2)
a [Å]	11.6799(6)	11.8110(5)	9.634(6)	15.3526(11)	7.1928(4)	7.2417(2)	21.4585(12)	11.0607(7)
b [Å]	8.0262(4)	12.8065(5)	9.422(6)	15.6010(11)	13.8777(8)	11.2409(3)	12.2662(7)	11.0707(7)
c [Å]	13.2131(7)	18.3660(7)	21.150(13)	16.0353(12)	15.0416(9)	19.6167(6)	11.1763(6)	12.6727(8)
α [°]	90	90	90	90	90	90	90	114.8432(18)
β [°]	90	90	96.860(17)	94.2679(10)	90	91.9052(11)	90	93.5680(19)
γ [°]	90	90	90	90	90	90	90	94.7534(19)
Volume [$Å^3$]	1238.66(11)	2778.00(19)	1906(2)	3830.1(5)	1501.45(15)	1595.98(8)	2941.8(3)	1395.22(15)
Z	4	8	4	4	4	4	4	2
ρ_{calc} [gcm^{-3}]	2.375	2.242	2.667	2.382	2.235	2.120	2.251	2.256
μ [mm^{-1}]	11.694	10.436	15.173	9.766	9.835	9.126	9.876	10.398
F(000)	832	1776	1408	2552	960	968	11901	896
Crystal size [mm^3]	0.180×0.100×0.10	0.100×0.090×0.04	0.12×0.09×0.05	0.150×0.020×0.013	0.150×0.080×0.08	0.180×0.070×0.06	0.17×0.11×0.01	0.310×0.120×0.030
Crystal colour	colourless	bronze	purple	bronze	yellow	yellow	colourless	Colourless
Crystal shape	fragment	needle	block	fragment	block	needle	plate	Irregular
Radiation	MoK_α (λ=0.71073 Å)	MoK_α (λ=0.71073 Å)	MoK_α (λ=0.71073 Å)	MoK_α (λ=0.71073 Å)	MoK_α (λ=0.71073 Å)	MoK_α (λ=0.71073 Å)	MoK_α (λ=0.71073 Å)	MoK_α (λ=0.71073 Å)
2Θ range [°]	5.94 to 60.09 (0.71 Å)	4.44 to 60.28 (0.71 Å)	3.88 to 56.82 (0.75 Å)	3.65 to 54.28 (0.78 Å)	3.99 to 60.14 (0.71 Å)	4.15 to 65.43 (0.66 Å)	3.80 to 52.88 (0.80 Å)	3.72 to 54.25 (0.78 Å)
Reflections collected	21524	42495	68330	71636	50835	78514	82510	49640
Independent reflections	3560 R_{int} = 0.0134 R_{sigma} = 0.0099	4105 R_{int} = 0.0259 R_{sigma} = 0.0123	4745 R_{int} = 0.0343 R_{sigma} = 0.0136	8483 R_{int} = 0.0676 R_{sigma} = 0.0390	4405 R_{int} = 0.0433 R_{sigma} = 0.0244	5862 R_{int} = 0.0336 R_{sigma} = 0.0169	6034 R_{int} = 0.0730 R_{sigma} = 0.0347	6173 R_{int} = 0.0566 R_{sigma} = 0.0329
Completeness	100.0 %	100.0 %	100.0 %	100.0 %	100.0 %	100.0 %	100.0 %	100.0 %
Data / Restraints / Parameters	3560/1/146	4105/0/173	4745/5/218	8483/48/448	4405/2/178	5862/2/196	6034/148/374	6173/4/331
Goodness-of-fit on F^2	1.038	1.023	1.276	1.050	1.020	1.033	1.088	1.006
Final *R* indexes [$I \geq 2\sigma(I)$]	R_1 = 0.0086 wR_2 = 0.0187	R_1 = 0.0119 wR_2 = 0.0226	R_1 = 0.0451 wR_2 = 0.1075	R_1 = 0.0261 wR_2 = 0.0599	R_1 = 0.0152 wR_2 = 0.0309	R_1 = 0.0176 wR_2 = 0.0348	R_1 = 0.0397 wR_2 = 0.0887	R_1 = 0.0238 wR_2 = 0.0509
Final *R* indexes [all data]	R_1 = 0.0091 wR_2 = 0.0189	R_1 = 0.0168 wR_2 = 0.0235	R_1 = 0.0470 wR_2 = 0.1082	R_1 = 0.0412 wR_2 = 0.0668	R_1 = 0.0176 wR_2 = 0.0314	R_1 = 0.0247 wR_2 = 0.0367	R_1 = 0.0531 wR_2 = 0.0961	R_1 = 0.0353 wR_2 = 0.0550
Largest peak/hole [$eÅ^{-3}$]	0.28/-0.28	0.43/-0.47	3.06/-3.19	1.95/-2.06	1.12/-0.73	1.49/-0.82	2.88/−1.42	1.68/-1.84
Flack X parameter	-0.017(2)				-0.001(3)		0.489(19)	
Extinction coefficient	0.00048(5)							

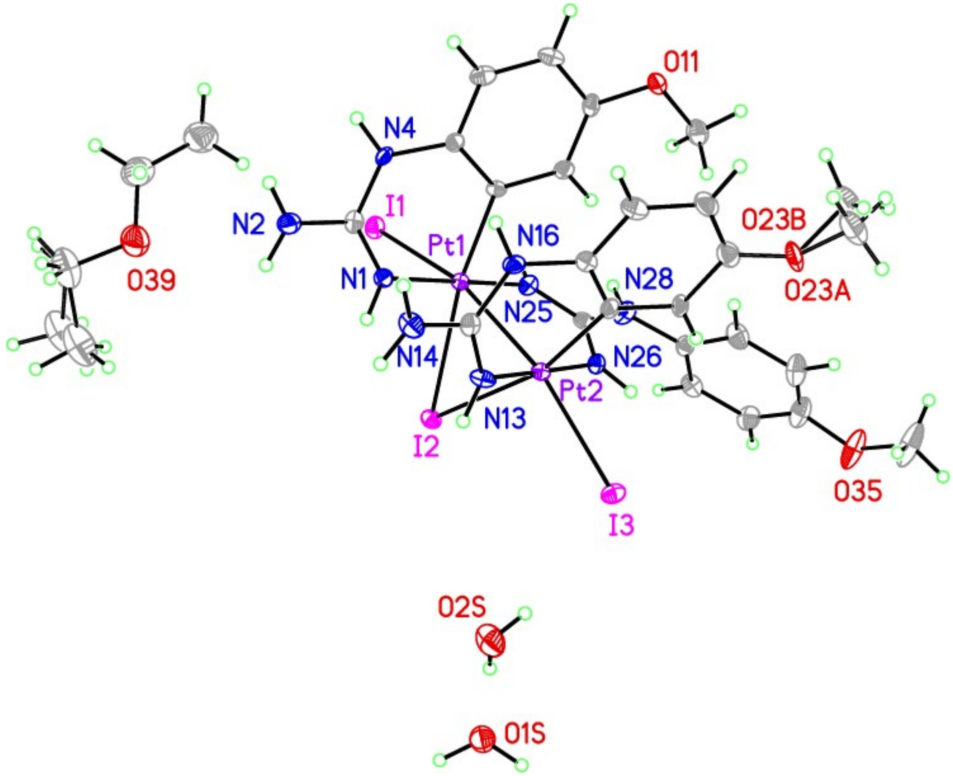

Figure S4. Molecular structure of **18** showing the disordered diethyl ether and water solvates. Disordered methoxy group also shown (O23,C24). Displacement parameters shown at 50% probability.

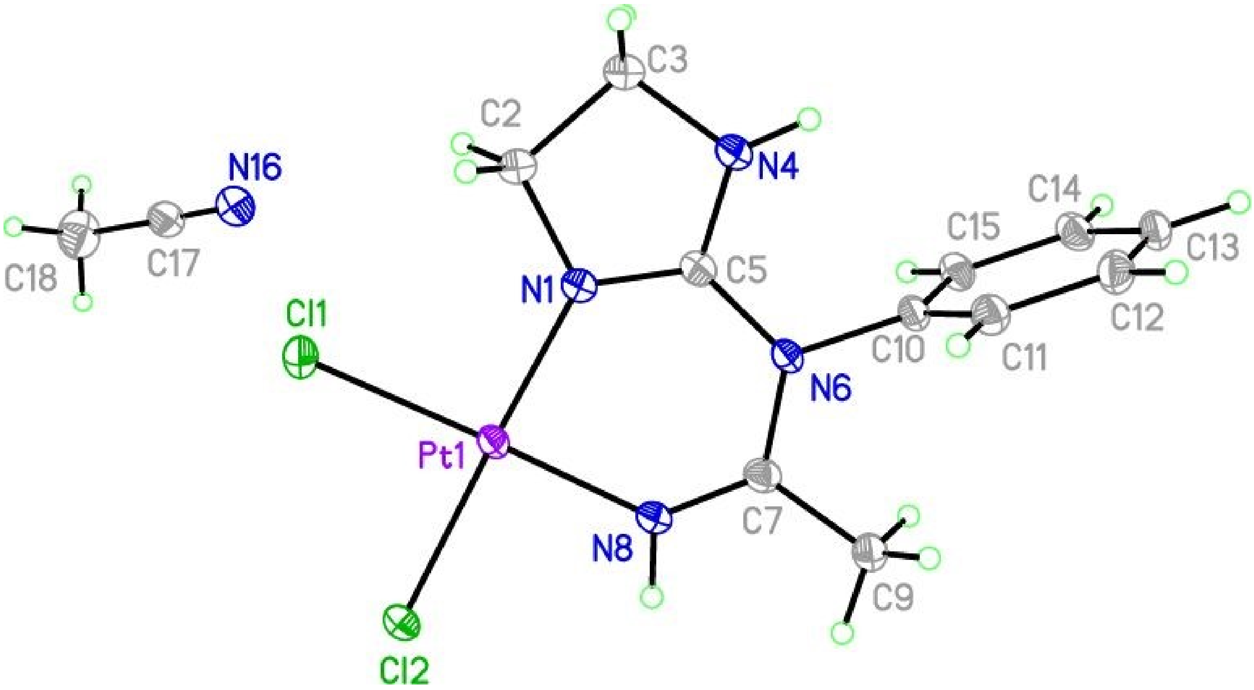

Figure S5. Complete asymmetric unit of **20** showing the acetonitrile solvates. Displacement parameters shown at 50% probability.

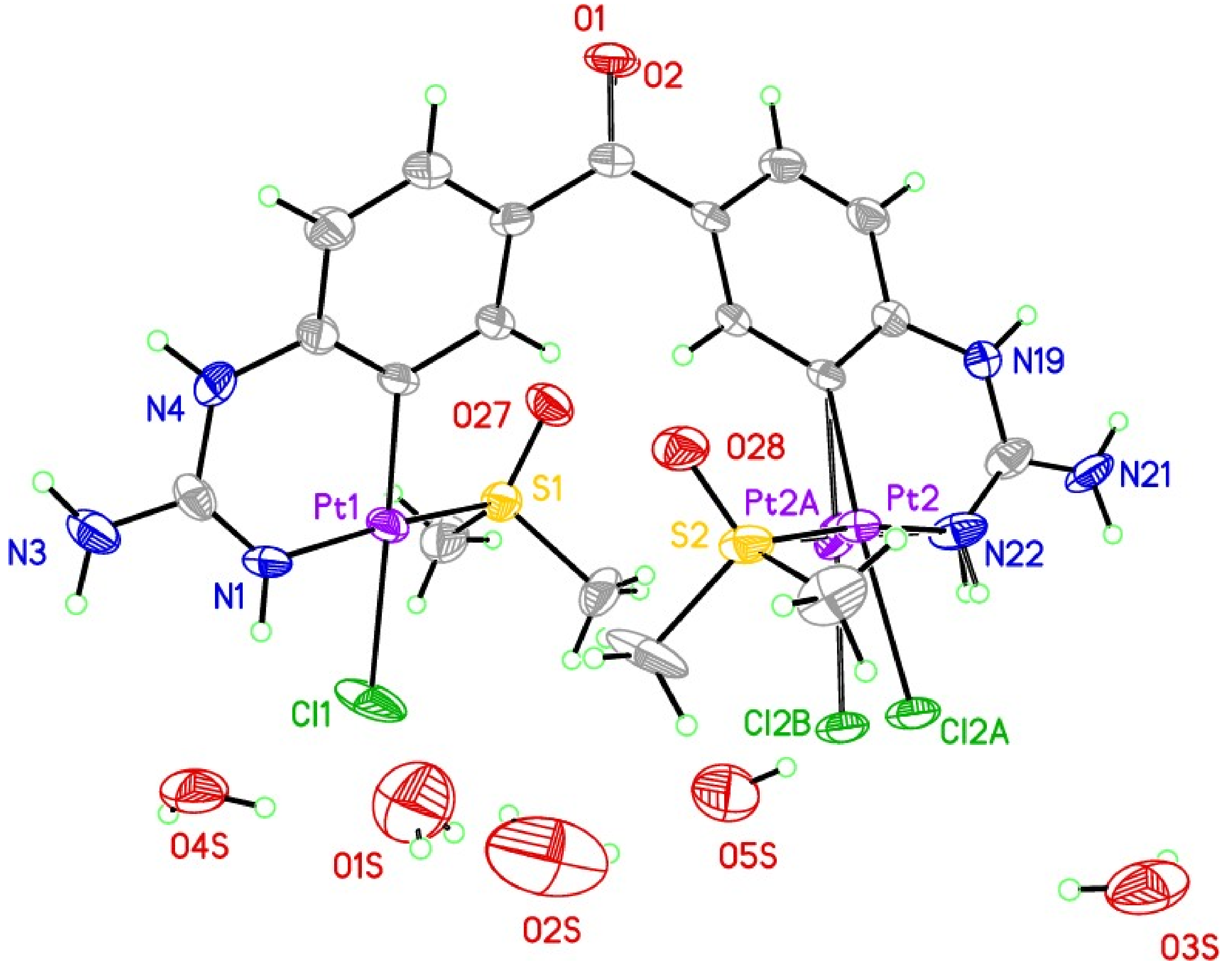

Figure S6. Complete asymmetric unit of **25i** showing disorder in the Pt2/Cl2 positions (72:28% occupied) and the water solvates. The occupancy of O5s is linked to Cl2a (72% occupied). Displacement parameters shown at 50% probability.

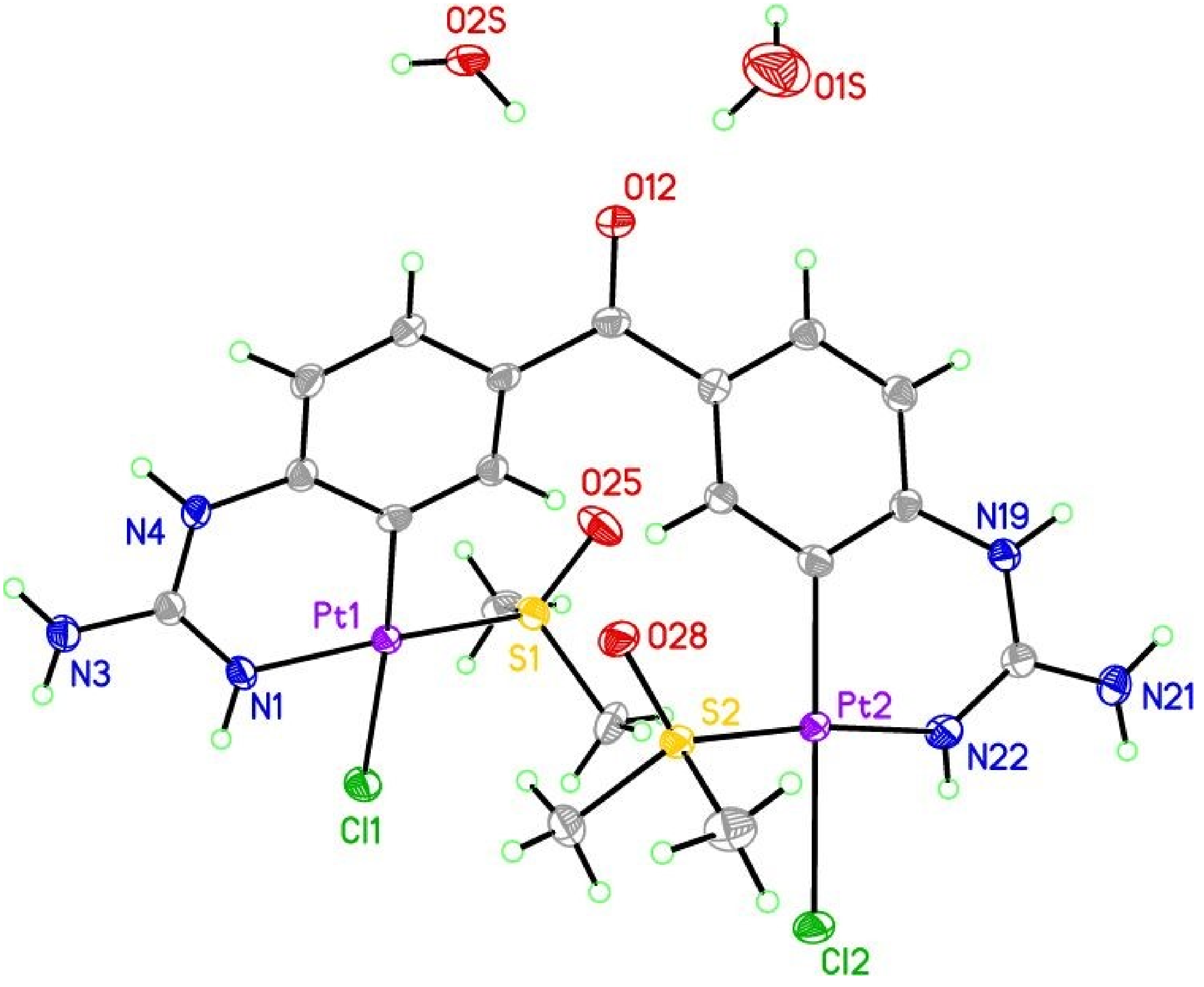

Figure S7. Complete asymmetric unit of **25ii** with water solvates O1s, O2s fully occupied. Displacement parameters shown at 50% probability.

References

1. F. Rodriguez, I. Rozas, J. E. Ortega, A. M. Erdozain, J. J. Meana and L. F. Callado, *J. Med. Chem.*, 2008, **51**, 3304–12.
2. F. Rodriguez, I. Rozas, J. E. Ortega, J. J. Meana and L. F. Callado, *J. Med. Chem.*, 2007, **50**, 4516–27.
3. B. Kelly, M. McMullan, C. Muguruza, J. E. Ortega, J. J. Meana, L. F. Callado and I. Rozas, *J. Med. Chem.*, 2015, **58**, 963–977.
4. Y. F. Yong, J. A. Kowwalski, J. C. Thoen, and M. A. Lipton, *Tet. Lett*. 1999, **40**, 53-56
5. B. Kelly and I. Rozas, *Tet. Lett.*, 2013, **54**, 3982–3984.
6. Bruker, *SAINT*, Bruker AXS Inc., Madison, Wisconsin, USA.
7. L. Krause, R. Herbst-Irmer, G. M. Sheldrick, D. Stalke, *J. Appl. Cryst.* **2015**, *48*, 3–10.
8. G. M. Sheldrick, *Acta Cryst.* **2015**, *A71*, 3–8.
9. G. M. Sheldrick, *Acta Cryst.* **2015**, *C71*, 3–8.
10. O. V. Dolomanov, L. J. Bourhis, R. J. Gildea, J. A. K. Howard, H. Puschmann, *J. Appl. Cryst.* **2009**, *42*, 339-341.
11. C. R. Groom, I. J. Bruno, M. P. Lightfoot, S. C. Ward, *Acta Cryst.* **2016**, *B72*, 171–179.
12. D. Kratzert, *FinalCif*, *V139*, https://dkratzert.de/finalcif.html.
